# Structured Connectivity in Cerebellar Inhibitory Networks

**DOI:** 10.1016/j.neuron.2013.12.029

**Published:** 2014-02-19

**Authors:** Sarah Rieubland, Arnd Roth, Michael Häusser

**Affiliations:** 1 Wolfson Institute for Biomedical Research and Department of Neuroscience, Physiology and Pharmacology, University College London, Gower Street, London WC1E 6BT, UK

## Abstract

Defining the rules governing synaptic connectivity is key to formulating theories of neural circuit function. Interneurons can be connected by both electrical and chemical synapses, but the organization and interaction of these two complementary microcircuits is unknown. By recording from multiple molecular layer interneurons in the cerebellar cortex, we reveal specific, nonrandom connectivity patterns in both GABAergic chemical and electrical interneuron networks. Both networks contain clustered motifs and show specific overlap between them. Chemical connections exhibit a preference for transitive patterns, such as feedforward triplet motifs. This structured connectivity is supported by a characteristic spatial organization: transitivity of chemical connectivity is directed vertically in the sagittal plane, and electrical synapses appear strictly confined to the sagittal plane. The specific, highly structured connectivity rules suggest that these motifs are essential for the function of the cerebellar network.

## Highlights

• Quadruple patch recordings from interneurons reveal highly nonrandom connectivity
• Both chemical and electrical interneuron networks exhibit spatial clustering
• Chemical synaptic connections are organized in transitive feedforward motifs
• The electrical and chemical networks show specific overlap at the triplet level

## Teaser

Interneurons are connected by both chemical and electrical synapses, but the organization of this connectivity is unknown. With quadruple recordings from cerebellar interneurons, Rieubland et al. find that electrical and chemical interneuron networks exhibit highly structured nonrandom connectivity, including clustered and feedforward motifs.

## Introduction

Neural circuits are the substrate for information processing and behavior. However, little is known about the rules governing their connectivity and the motifs they form in the mammalian brain. Identifying such rules and motifs is important, because the fine structure of connectivity influences activity patterns, information processing, and memory storage in neural circuits (Denk et al., 2012; Seung, 2009). Although the large-scale connectivity between brain areas is evidently structured, it has been proposed that local connectivity between individual cells may be random, and mostly governed by spatial constraints. In particular, cortical connectivity has been proposed to result from nonspecific overlap between axons and dendrites, the so-called Peters’ rule (Braitenberg and Schüz, 1991; Peters and Feldman, 1976). Because the concept of randomly connected neural networks constitutes one of the simplest assumptions, it has been widely used for network models and theory (Markram, 2006).

However, evidence has recently emerged in favor of structured local circuits. The *C. elegans* connectome has been shown to contain small-world properties (Watts and Strogatz, 1998) and specific functional motifs (Milo et al., 2002; Varshney et al., 2011). Many brain areas reveal signs of structured connectivity, in particular, in relation to their functional representation (Briggman et al., 2011; Helmstaedter et al., 2013; Ko et al., 2011; Maisak et al., 2013; Takemura et al., 2013). Connectivity inferred from neural activity at a scale of hundreds of neurons also suggests small-world properties (Yu et al., 2008) and the presence of hub neurons (Bonifazi et al., 2009). Other approaches for probing functional connectivity in a sparse manner also provide evidence for specific organization. These studies have investigated connectivity between principal cells of the same type (Ko et al., 2011; Perin et al., 2011; Song et al., 2005), where nonrandom features and clustering are present, and between different types of principal cells, where cortical layer specificity governs connectivity (Kampa et al., 2006; Lefort et al., 2009; Yoshimura et al., 2005). The connectivity between interneurons and principal cells has also been explored especially in the neocortex, where the large diversity of interneuron types suggests functional diversity. These studies generally report a cell-type-specific organization between cortical layers (Jiang et al., 2013; Kätzel et al., 2011; Yoshimura and Callaway, 2005; Yoshimura et al., 2005), but a dense nonspecific local connectivity (Fino and Yuste, 2011; Packer and Yuste, 2011). The connectivity from excitatory to inhibitory cells (Bock et al., 2011; Hofer et al., 2011) suggests that cortical interneurons sample their excitatory inputs randomly. The available results thus indicate that interconnectivity of principal cells is structured, whereas connectivity of interneurons is unstructured. However, an important element remains to be probed in more detail: the higher-order connectivity among interneurons. Recently, the interaction between the different types of cortical interneurons and its functional implications have attracted interest (Jiang et al., 2013; Letzkus et al., 2011; Pi et al., 2013). Interneuron networks are known to share electrical and/or chemical synapses in various brain areas (Bartos et al., 2002; Galarreta and Hestrin, 1999, 2002; Gibson et al., 1999; Landisman et al., 2002; Tamás et al., 2000), including in a cell-type-specific manner (Blatow et al., 2003; Gibson et al., 1999; Jiang et al., 2013; Koós and Tepper, 1999) and are thought to underlie important features of network dynamics, such as synchronization and oscillations (Bartos et al., 2007; Whittington and Traub, 2003). However, quantitative information about the connectivity motifs and network architecture of interneuron-interneuron connections, in particular among interneurons of the same cell type, is still lacking and is essential in order to fully understand their operation (Buzsáki et al., 2004).

Molecular layer interneurons in the cerebellum play an important role in regulating cerebellar output and motor learning (Jörntell et al., 2010). They are interconnected by GABAergic chemical synapses (Häusser and Clark, 1997; Llano and Gerschenfeld, 1993) and by electrical synapses (Alcami and Marty, 2013; Mann-Metzer and Yarom, 1999). The connections between molecular layer interneurons have important functional roles: the electrical connections can promote synchrony (Mann-Metzer and Yarom, 1999), whereas the chemical synapses can delay action potentials and affect the precision of spike timing in postsynaptic interneurons (Häusser and Clark, 1997; Mittmann et al., 2005). However, the level of overlap between the chemical and electrical networks and their higher-level organization remain unclear.

Here, we use multiple whole-cell patch-clamp recordings to investigate the electrical and chemical connectivity of the interneuron network in the molecular layer of the cerebellum. We find that the structure of the network follows mostly random connectivity predictions at the level of pairs of neurons but deviates strongly from these predictions when probed at the level of triplets and quadruplets of neurons. Chemical synapses preferably form transitive connectivity motifs, such that if cell A connects to cell B, and B to C, then cell A also connects to cell C. We show that the observed connectivity is supported by a defined spatial organization: electrical synapses are restricted to sagittal planes, and the chemical transitivity is oriented in the sagittal plane. These signs of structured connectivity have important implications for the function of the network.

## Results

### Overlap of Electrical and Chemical Networks in Molecular Layer Interneurons

**Figure 1:**
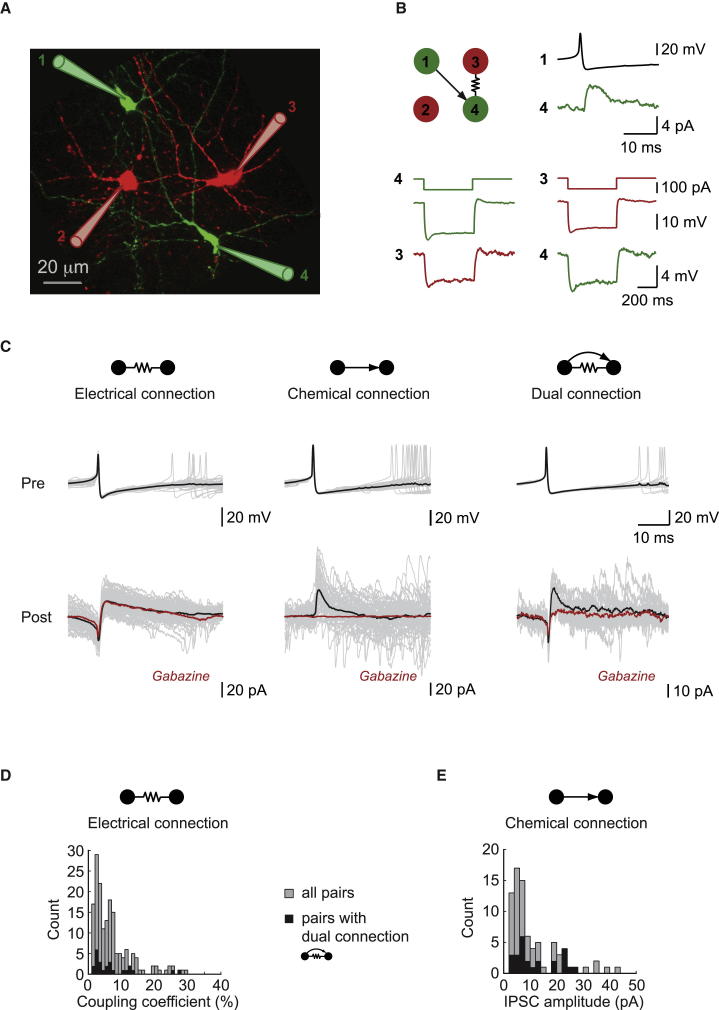
Molecular Layer Interneurons Are Connected by Electrical and Chemical Synapses (A) Simultaneous whole-cell patch-clamp recording from four molecular layer interneurons (MLI), filled with Alexa 488/594 and imaged with two-photon microscopy. (B) Testing for functional connections reveals an inhibitory chemical connection between cells 1 and 4 and an electrical connection between cells 3 and 4. (C) Examples of the three types of connections observed in voltage clamp before and after gabazine (SR95531) application. Traces are spike-triggered averages (>30 sweeps). (D and E) Distribution of synaptic strengths across the population (gray) and for dual connections (black): coupling coefficient for electrical connections (D) and IPSC amplitude (at VC = −50 mV) for chemical connections (E).

We used multiple simultaneous patch-clamp recordings (Figure 1A) to assess the connectivity among molecular layer interneurons (MLIs) in rat cerebellar slices (P18–23). MLIs are connected by GABAergic synaptic connections (Häusser and Clark, 1997; Kondo and Marty, 1998), and by electrical coupling via gap junctions (Alcami and Marty, 2013; Mann-Metzer and Yarom, 1999). We therefore investigated the extent of overlap between these two populations.

Electrical coupling between individual pairs of neurons was quantified with long current pulses (Figure 1B), and the coupling coefficient (CC) of the connection was determined (Supplemental Experimental Procedures available online). The postsynaptic voltage response to a spontaneous action potential (AP) in an electrically coupled presynaptic cell consisted of a spikelet (0.30 ± 0.42 mV, n = 77; for CC ≥1%) followed by an afterhyperpolarization (AHP; 0.46 ± 0.58 mV, n = 77), as observed between other coupled cells with large AHPs (Galarreta and Hestrin, 2002; Vervaeke et al., 2010; Figure S1). In voltage clamp, the postsynaptic current corresponds to the inverted, filtered presynaptic AP (Figure 1C, left). The mean CC of electrically coupled pairs was 7.13% ± 6.02% (n = 171), although it spanned a wide range, with a few CCs being over 25% Figure 1D). The overall probability of finding an electrical connection at the pair level was p_E_ = 0.42.

The presence of chemical synapses was tested by examining the average synaptic current evoked in the postsynaptic cell by a presynaptic AP (Figure 1B). Purely GABAergic chemical synaptic connections were characterized by an outward inhibitory postsynaptic current (IPSC) (when holding at −50 mV) that was completely abolished by 10 μM gabazine (SR95531; Figure 1C, middle). The mean IPSC amplitude was 11.2 ± 9.2 pA (n = 80; Figure 1E). The overall probability of observing a chemical connection was p_C_ = 0.20, whereas the probability of a given pair being connected with at least one chemical synapse (unidirectional or bidirectional) was p = 0.36.

A significant proportion of MLI pairs were connected via both chemical and electrical synapses, which we term “dual connections,” as found in other brain regions (Galarreta and Hestrin, 2002; Tamás et al., 2000). At such connections, the electrical synapse can be detected using hyperpolarizing current pulses. The postsynaptic response to a presynaptic AP will, however, consist of a mixture of the GABAergic synaptic current and the filtered electrically coupled AP. These can be disentangled by applying gabazine, which blocks the GABAergic IPSC and isolates the remaining electrical component (Figure 1C, right). In contrast, a pure electrical response is unaffected by gabazine application (Figure 1C, left). The distribution of synaptic strengths for the electrical and chemical components of dual connections was similar to that of the overall population (Figures 1D and 1E). The overall probability of dual connections was p_D_ = 0.12. These results show that the chemical and electrical networks within the interneuron population of the cerebellar molecular layer can overlap.

### Distance Dependence of Electrical and Chemical Connection Probability

**Figure 2:**
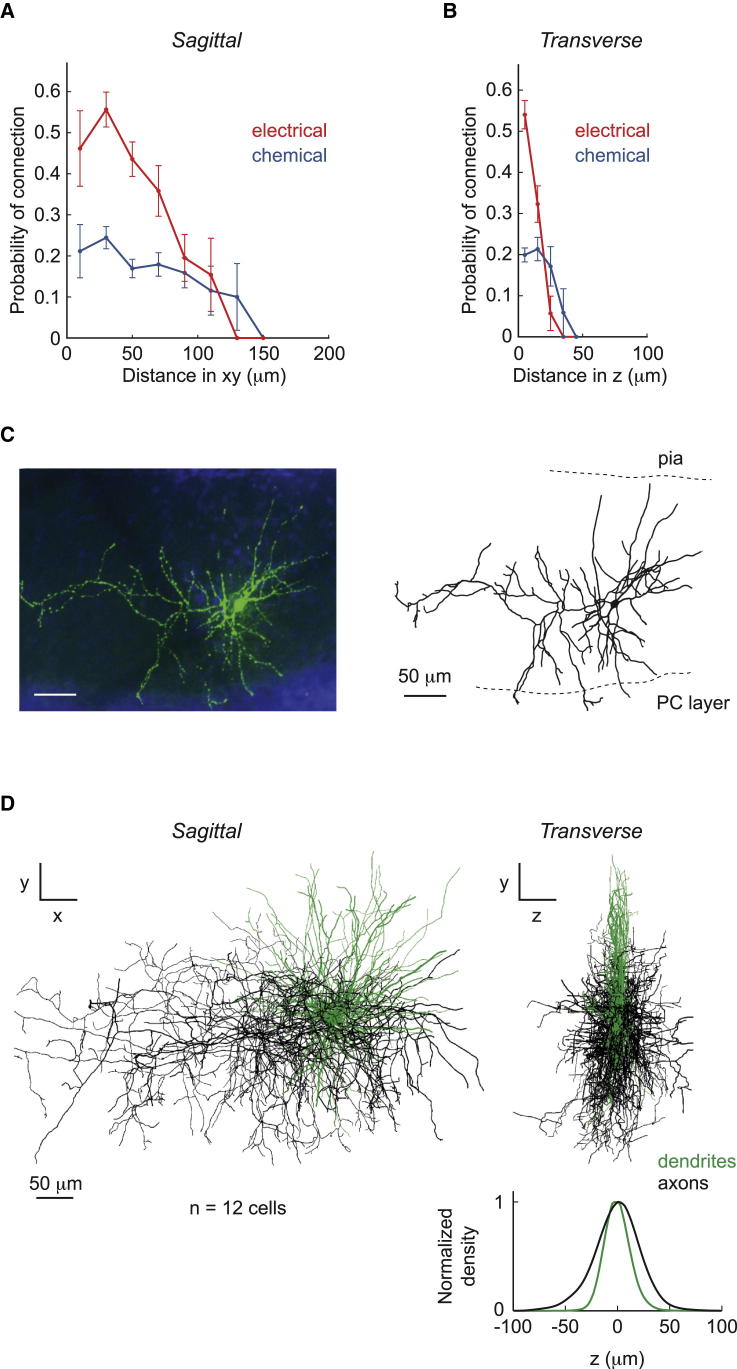
Distance Dependence of Electrical and Chemical Connection Probability (A) Probability of electrical and chemical connections versus intersomatic distance in xy (sagittal plane) between recorded pairs. (B) Probability of electrical and chemical connections versus intersomatic distance in z (transverse axis) between recorded pairs. Error bars indicate SD based on bootstrap analysis. (C) MLI filled with biocytin and imaged using confocal microscopy after streptavidin-conjugated Alexa 488 histochemistry (left; blue, DAPI), and its reconstructed morphology (right). (D) Superposition of 12 reconstructed MLI morphologies in xy view (left) and yz view (right). Bottom right, normalized density profile along the z axis. Dendrites (green) are more strongly confined to the sagittal plane than axons (black).

We next examined how the probability of connections between individual MLI pairs depends on the intersomatic distance, after confirming that our estimate of connection probability is not affected by the slicing process (Figure S2A). Over the distances tested (≤180 μm in the sagittal Δxy plane; ≤50 μm along the transverse Δz axis; Figures S2B and S2C), the probability of an electrical connection p_E_ and chemical connection p_C_ decreased with both increasing Δxy and Δz (Figure 2A). Along the transverse axis, the electrical coupling appears confined to a remarkably narrow plane, with Δz ≤30 μm (Figure 2B), whereas the chemical connection is less strongly confined.

These results can be explained by the somatodendritic morphology of MLIs: their dendrites are planar and follow the sagittal plane, similarly to Purkinje cell dendrites (Palay and Chan-Palay, 1974; Rakic, 1972; Sultan and Bower, 1998), whereas their axons have a broader spatial distribution. To quantify the difference between the spatial extent of axons and dendrites, we reconstructed MLIs individually filled with biocytin and imaged their structure using high-resolution confocal microscopy (Figure 2C). Their morphologies were centered and realigned with respect to the sagittal plane and pial surface (Supplemental Experimental Procedures; Figure 2D; n = 12 cells) and used to generate a density map in the xy and yz planes. The width of the normalized density map of dendrites and axons along the z axis was estimated as 2σ (dendrite) = 24.1 μm and 2σ (axon) = 41.3 μm, respectively (Figure 2D, right). Thus, dendrites are more segregated to the sagittal plane than axons, which, given the dendritic location of electrical synapses between MLIs (Sotelo and Llinás, 1972), explains the tighter spatial confinement of electrical coupling.

### Connectivity at the Pair Level Appears Mostly Random

**Figure 3:**
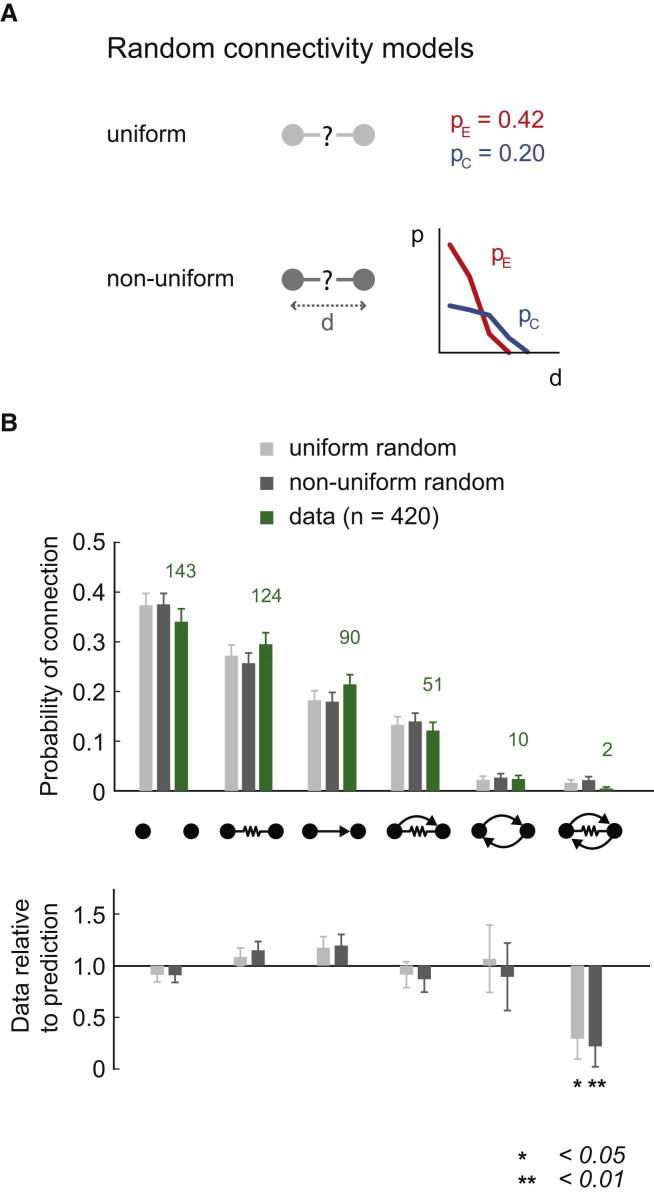
Connectivity at the Pair Level Appears Mostly Random (A) Comparing the predictions of random connectivity models to experimental data. The uniform random prediction is based on the average unidirectional connection probabilities (light gray). The nonuniform random prediction is based on the intersomatic distance and the measured probabilities of connections as a function of distance (dark gray). (B) Probability of each type of connection between pairs: no connection, electrical only, chemical only, dual, bidirectional, and bidirectional and electrical, compared to the two predictions. For each connection type, the number of observations in the experiment is given above the green bars.

Is the connectivity between interneurons random on the level of individual pairs? To answer this question, we first calculated the overall probabilities for each type of connection between pairs in the data. The probability of a pair having no chemical or electrical connection was p = 0.340; electrical only p = 0.295; chemical only p = 0.214; dual chemical and electrical p = 0.121; bidirectional chemical p = 0.024; and bidirectional chemical with electrical p = 0.005. To test whether these results are consistent with the null hypothesis (“connectivity is random”), it was necessary to generate synthetic connectivity data defined as random and compare it to the real data. Any significant difference would disprove the null hypothesis and show nonrandom features of connectivity. We can formulate two sets of predictions for the pairwise connection probabilities, both based on random statistics. The first one only assumes that all chemical and electrical connections are made independently of each other with the average connection probabilities p_E_ = 0.42 and p_C_ = 0.20 (Figure 3A, top; Supplemental Experimental Procedures). It represents a simple model of locally uniform random synaptic connectivity between pairs of cells. We name this first model the “uniform random” model. The second, more complex model also assumes that all connections are made independently of each other, but the probability of a connection depends on the intersomatic distance in xy and z (Figure 3A, bottom). We constructed the model of distance dependence using the distributions observed in the data (Figures 2A, 2B, S2D, and S2E). We call this second model the “nonuniform random” model. In addition, we also tested two random models that include the position of the cells in the molecular layer (ML) as a parameter (Figure S3). The probabilities of the different connection types between pairs predicted by the two models (Figure 3B; light and dark gray bars) were compared to the data (green bars, n = 420 pairs). For most of the connection types the ratio of the predicted to the actual connection probability is not significantly different from 1. The occurrence of fully connected (bidirectional chemical and electrical) pairs is significantly lower than predicted by both random models (p = 0.046 and 0.004 for the uniform and nonuniform random predictions, respectively; though the difference is not significant when including ML position in the random model, Figures S4A and S4B). The occurrence of bidirectional chemical connections at the random level is in contrast to excitatory connections between layer 5 pyramidal cells, where they are overrepresented (Markram et al., 1997; Song et al., 2005; Perin et al., 2011). In addition, the number of dual connections is at the level expected if electrical and chemical synapses are formed independently of each other. Thus, the fact that only small differences were observed compared to the predictions appears to suggest that random connectivity is an adequate model at the pair level for these interneuron networks.

### Stronger Electrical Clustering Than Predicted by the Random Connectivity Models

**Figure 4:**
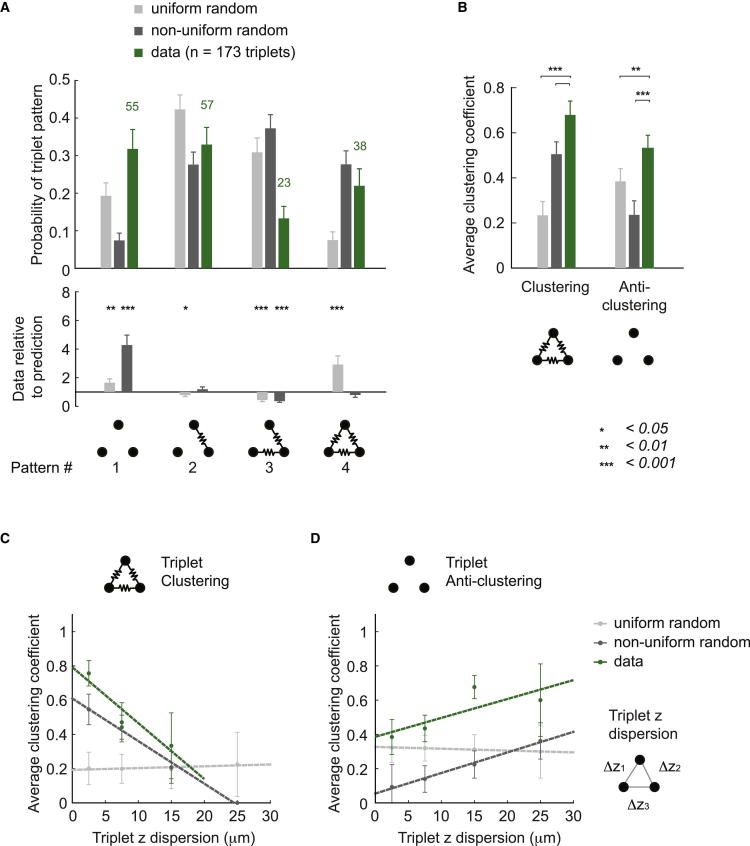
The Electrical Network Exhibits Clustering in the Sagittal Plane (A) Probability of observing each of the four nonisomorphic triplet motifs of electrical connections (n = 173 triplets) compared to uniform random and nonuniform random predictions. (B) Average clustering *C* and anticlustering coefficient *AC* of triplets and quadruplets for electrical connections compared to both predictions. (C) Average clustering coefficient of triplets versus their mean z dispersion for the data and for the two predictions (linear fits). (D) Average anticlustering coefficient of triplets versus their mean z dispersion for the data and for the two predictions (linear fits).

We next examined connectivity motifs involving more than two neurons. To address this, we investigated the higher-order connectivity among triplets and quadruplets of neurons and compared the findings to the two random connectivity models. First, we counted the occurrences of each possible electrical triplet pattern (Figure 4A). The recorded quadruplets were separated into triplets for a total of n = 173 triplets. The intersomatic distances measured for each configuration were used to predict the probability of electrical and chemical connections for the nonuniform random model. The occurrences predicted by both random models were counted in the same way as for the data (Supplemental Experimental Procedures). The ratio (data/prediction) indicates the relative occurrence of each of the four possible nonisomorphic patterns, compared to the two random connectivity predictions (Figure 4A).

We found that the predictions of both random connectivity models differ from the data. The uniform random prediction shows large deviations compared to the data for most patterns (p values: p_1_ = 0.003, p_2_ = 0.022, p_3_ = 0.0004, p_4_ = 0.0004), confirming that the model is insufficient to describe the statistics of connections of the MLI network. The nonuniform random prediction also deviates from the data but to a lesser degree, as the occurrence of fully connected triplets (pattern 4) is correctly predicted (p values: p_1_ = 0.0004, p_2_ = 0.213, p_3_ = 0.0004, p_4_ = 0.202). We separately confirmed that the fully interconnected triplets (pattern 4) are indeed the result of direct connections and not indirect electrical coupling (Figure S4E).

To characterize the electrical connectivity with a single measure and compare it to random connectivity models, we used the clustering coefficient *C*. *C* was originally introduced as a measure of the topological organization of networks and used to highlight differences between small-world networks and random networks, whose average *C* are significantly different (Watts and Strogatz, 1998). *C* is usually measured for each node in a network. Here, we calculate *C* for the recorded subnetworks of triplets and quadruplets of MLIs and compute the average over the configurations where *C* could be measured (Supplemental Experimental Procedures). It should be noted that the average *C* obtained in this way is not intended to represent the average *C* of the whole network but is used to compare with *C* predicted by random connectivity models, where it was also calculated for subnetworks of triplets and quadruplets. For triplets, *C* effectively measures the likelihood that if neurons A and B, and B and C are connected, then A and C are also connected.

The nonuniform random model predicted a higher clustering coefficient for electrical synapses, *C*_E_, than the uniform random model. This is expected if the tested neurons are sampled locally, as they were in the experiments (Figures S2B and S2C). However, *C*_E_ of the data significantly exceeds even the nonuniform random prediction (Figure 4B; uniform random p = 0.0001; nonuniform random p = 0.0001). Thus, whereas the nonuniform random model provides an improved prediction compared to the uniform random model, it is still not sufficient to accurately describe the connectivity of MLIs. The remaining difference can be mainly explained by the underrepresentation of triplets with two connections (Figure 4A, pattern 3, *C*_E_ = 0), highlighting the relevance of predicting the absence of connections in random connectivity models.

To further explore the importance of the absence of connections, we examined the anticlustering coefficient (*AC*), which is calculated in the same way as the *C* but using the complement graph (Supplemental Experimental Procedures). It measures the likelihood that if neurons A and B as well as B and C are not connected, then A and C are not connected either. We found a higher *AC*_E_ in the data compared to the nonuniform random prediction (Figure 4B; uniform random p = 0.005; nonuniform random p = 0.0001), which is due to the overrepresentation of unconnected triplets in the data (Figure 4A; pattern 1, *AC*_E_ = 1). To summarize, the random connectivity models do not correctly represent the clustering and anticlustering of the MLI subnetworks because they do not correctly predict the absence of connections in a triplet.

Finally, we investigated how *C*_E_ and *AC*_E_ are related to the spatial arrangement of neurons in the network, in particular, along the transverse axis, given that electrical connections appear confined to an ∼20 μm thick layer (Figure 2B). For each triplet, we used the dispersion in the transverse axis (the mean of Δz for each connection; Figures 4C and 4D), and, as expected, the uniform random prediction yields a constant *C*_E_ and *AC*_E_ value. The *C*_E_ for the data decreases rapidly with larger z dispersion of the triplet (linear fit, slope = −0.033/μm, y intercept = 0.79), which is predicted by the nonuniform random model with a lower slope and a significantly lower y intercept (slope = −0.025/μm, y intercept = 0.61; p = 1.9 × 10^−6^; Figure 4C). The *AC*_E_ for the data increases with larger z dispersion (slope = 0.011/μm, y intercept = 0.39), showing a significantly higher y intercept than the nonuniform random model prediction (slope = 0.012/μm, y intercept = 0.054; p = 1.5 × 10^−10^; Figure 4D). This shows that the nonuniform random model is not sufficient to explain the spatial organization of electrical connectivity, despite an improvement compared to the uniform random model.

### Transitive Chemical Motifs Are Overrepresented

**Figure 5:**
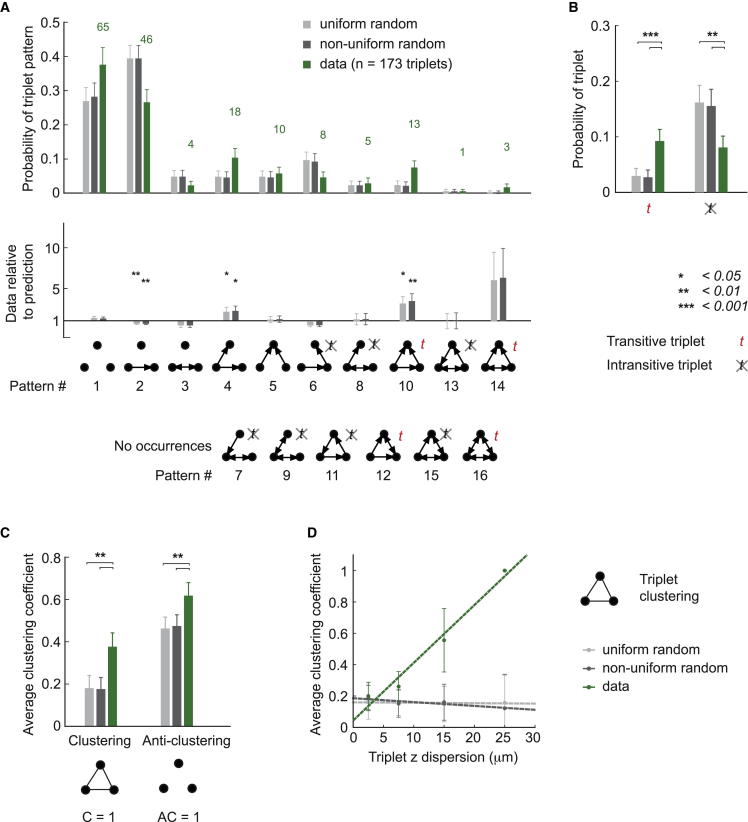
The Chemical Network Exhibits Transitive and Clustered Motifs across Sagittal Planes (A) Probability of observing each of the 16 nonisomorphic triplet motifs of chemical connections (n = 173 triplets) compared to uniform random and nonuniform random predictions. Motifs that did not occur in the data are presented at the bottom. (B) Probability of observing transitive patterns (marked *t* in A) and intransitive patterns, compared to predictions. (C) Average clustering and anticlustering coefficients of triplets and quadruplets for chemical connections compared to uniform random and nonuniform random predictions. (D) Average clustering coefficient of triplets versus their mean z dispersion for the data and the two predictions (linear fits).

To explore the higher-order connectivity of the chemical network, we next investigated individual chemical triplet patterns to identify which motifs are over- and underrepresented, using the same procedure as for the electrical triplets. In this case, it requires distinguishing uni- and bidirectional chemical connections, but not isomorphic triplet patterns, leading to 16 possible patterns (Supplemental Experimental Procedures; Figures 5A and S5A). For three patterns (patterns 2, 4, and 10), we found that the predictions of both random connectivity models were significantly different from the data. The triplet with a single connection or “directed edge” (pattern 2) is weakly underrepresented (ratio = 0.7 for both uniform and nonuniform random models; p = 0.0016 and 0.0064, respectively), the triplet with diverging connections or “V-out” (pattern 4) is overrepresented (ratio = 2.2 and 2.3 for the uniform and nonuniform random models; p = 0.043 and 0.022, respectively). The “feedforward” (pattern 10) is highly overrepresented (ratio = 3.2 and 3.5; p = 0.014 and 0.002, respectively).

Transitivity means that if there is a connection from cell A to cell B, and from cell B to cell C, there will also be a connection from A to C. Because a preference for transitive connectivity has been reported in other complex networks (Holland and Leinhardt, 1970; Milo et al., 2004), we tested this hypothesis in the MLI network and therefore grouped the patterns according to their property of transitivity (Bang-Jensen and Gutin, 2008; Supplemental Experimental Procedures; Figure S5A). Indeed, we found that intransitive patterns tend not to be observed in the data (e.g., the “three-loop” pattern 11, and the “mutual in” pattern 7), or appear to be underrepresented (the “three-chain” pattern 6, ratio = 0.5 compared to prediction of the nonuniform random model), whereas transitive patterns (e.g., the feedforward pattern 10, and the “regulating mutual” pattern 14) tend to be overrepresented (ratio = 3.5 and 6.3 compared to the prediction of the nonuniform random model). We therefore divided the observed patterns into two groups: transitive and intransitive. By this definition, patterns 10, 12, 14, 16 are transitive, and patterns 6, 7, 8, 9, 11, 13, 15 are intransitive (Figure 5A). Patterns 1, 2, 3, 4, 5 are excluded, as the property is not applicable due to the low number of connections. We observed significantly more transitive and significantly fewer intransitive patterns compared to both predictions (Figure 5B; uniform random: p = 0.0001 and 0.0016, respectively; nonuniform random: p = 0.0001 and 0.0026, respectively). This result highlights that random connectivity models are not sufficient to describe the connectivity of the MLI network, in particular, with respect to their transitive property.

To confirm the large deviation of the data compared to both models, we next calculated the average chemical clustering coefficient *C*_C_ and anticlustering coefficient *AC*_C_ for triplets and quadruplets, treating bidirectional and unidirectional connections identically. We observed a higher clustering coefficient *C*_C_ in the data than predicted by both random connectivity models (Figure 5C; p = 0.0020 and 0.0023, respectively). The values of *C*_C_ for the uniform random and nonuniform random predictions are similar due to the weak distance dependence of the probability of chemical connections (Figures 2A, 2B, and S2). We also found that *AC*_C_ was not correctly predicted by the random connectivity models (Figure 5C; p = 0.0012 and 0.0028, respectively). Together, these results indicate that the chemical connections are more clustered than predicted by the random connectivity models.

We next investigated the relationship between *C*_C_ and the dispersion of patterns along the transverse axis. Interestingly, we found that *C*_C_ increased at larger z dispersions (Figure 5D; linear fit, slope = 0.037/μm). This behavior strongly differs from both random connectivity predictions, which exhibit a mostly constant *C*_C_ (p = 3.3 × 10^−5^). This result means that the neurons in the triplet patterns with high *C*_C_ values can be on different sagittal planes distributed across the transverse axis. In conclusion, the chemical network has more clustered and transitive features than both random connectivity models predict and shows signs of spatial specificity.

### Structured Overlap between Electrical and Chemical Networks

After demonstrating the existence of structured features in the electrical and chemical networks, we investigated the overlap of the two networks. Because the number of potential individual mixed triplet patterns is very large (n = 128), we instead performed a common neighbor analysis (Perin et al., 2011). This is a method for investigating higher-order connectivity, and, in this case, the relationship between different connection types. It examines the effect of a common connected neighbor on the probability of connections of a given pair. We compared three probabilities: first, measured between pairs that have a common neighbor; second, measured between all other pairs (with no recorded common neighbor); and finally, predicted by the nonuniform random model, based on the distance between the pairs with common neighbor (and predicted by the nonuniform random model with ML position; Figure S6). The first comparison (pairs with common neighbor and all other pairs) offers an assessment of the higher-order structure within the data without the use of an explicit model of connectivity, but only under the assumption of independent connection probabilities. To simplify, we restricted the pair probability types to three: no connection, electrical connection, and chemical connection.

**Figure 6:**
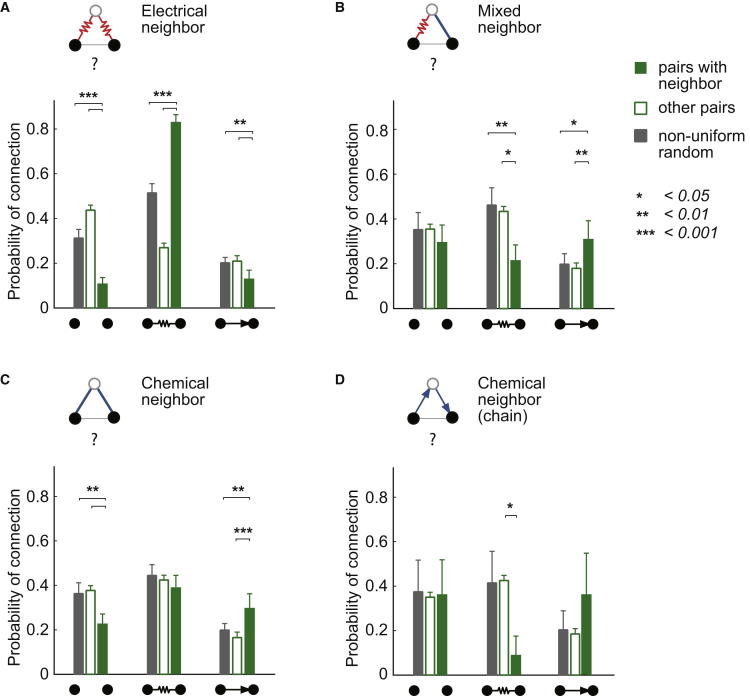
Common Neighbor Analysis Reveals Structured Overlap between Electrical and Chemical Networks (A–D) Connection probability between pairs sharing a common (electrical and/or chemical) neighbor, compared to other pairs and to the nonuniform random prediction. (A) Pairs sharing an electrical neighbor (n = 137). (B) Pairs sharing a mixed neighbor (electrical and chemical; n = 37). (C) Pairs sharing a chemical neighbor (any direction; n = 92). (D) Pairs sharing a chemical neighbor in a chain configuration (n = 11).

First, the presence of an electrical common neighbor (Figure 6A, n = 137) led to a higher probability of an electrical connection and a reduced probability of no connection compared to the other pairs (χ^2^ test, p = 7.39 × 10^−30^ and 1.11 × 10^−11^, respectively) and to the nonuniform random prediction (Monte Carlo, p = 0.0003 and 0.0003, respectively). This is consistent with the results shown in Figures 4A and 4B and confirms the preference for electrical clustered connectivity without the use of an explicit model of connectivity. Next, we examined the effect of a mixed (electrical and chemical) common neighbor on the pair connection probability (Figure 6B, n = 37). To test this independently of the preference for clustered electrical and chemical connectivity (Figures 6A and 6C), we excluded pairs with a common electrical neighbor and a common chemical neighbor from this analysis (the pairs can share a neighbor with one electrical and one chemical connection only). The presence of a mixed common neighbor resulted in a significantly lower probability of an electrical connection and a significantly higher probability of a chemical connection compared to other pairs (χ^2^ test, p = 0.016 and 0.003, respectively) and to the nonuniform random predictions (Monte Carlo, p = 0.0012 and 0.0296, respectively). This provides the first indication that the overlap between electrical and chemical connectivity is more structured than predicted by the random connectivity model. We then examined the effect of a chemical common neighbor, first disregarding the direction of the chemical connections (Figure 6C, n = 92). We observed an excess of chemical connections in these pairs compared to the other pairs and to the random model prediction (χ^2^ test, p = 1.39 × 10^−5^ and Monte Carlo, p = 0.0020), confirming the preference for fully connected chemical triplets, including the transitive ones seen in Figure 5A. Finally, we investigated the particular case of a common chemical neighbor in a chain configuration (Figure 6D, n = 11). This resulted in an underrepresentation of electrical connections compared to other pairs (χ^2^ test, p = 0.030; compared to the nonuniform random prediction p = 0.061). This result provides a second indication that the overlap between electrical and chemical networks is structured at the level of triplets of MLIs.

**Figure 7:**
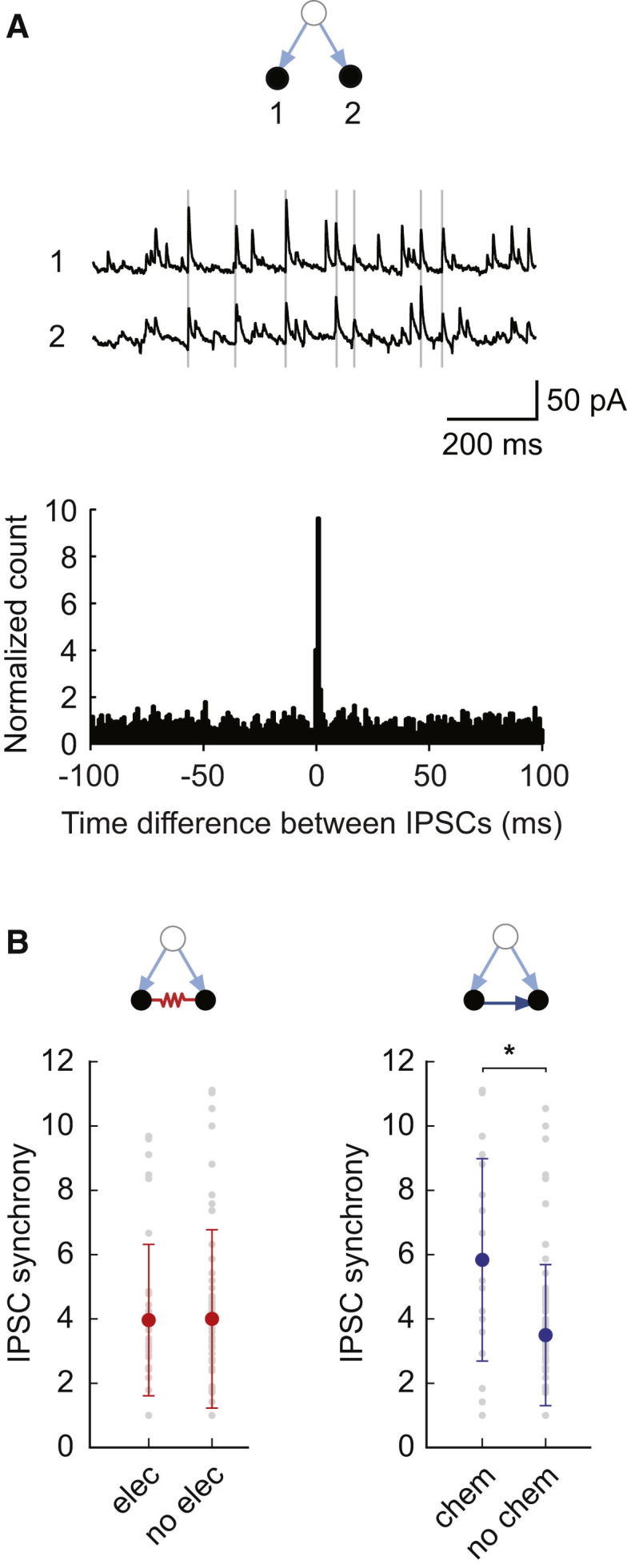
Functional Assay of Overlap between Electrical and Chemical Networks (A) Spontaneous IPSCs recorded in MLI pairs in VC = −50 mV. The peak of the normalized cross-correlogram (bin = 1 ms) defines the level of IPSC synchrony. (B) Level of IPSC synchrony between pairs with (n = 36) and without electrical connections (n = 50). Higher IPSC synchrony is observed between pairs with chemical connections (n = 18) than without (n = 68).

We next devised an independent way to obtain connectivity information from cells that were not directly recorded by measuring common synaptic inputs to a pair. This allows us to examine the configuration of diverging chemical connections made onto a pair of recorded neurons. The level of synchrony of IPSCs has been used previously as a measure for the likelihood of two neurons sharing a presynaptic partner (Sippy and Yuste, 2013; Vincent and Marty, 1993). We recorded spontaneous inhibitory input in simultaneously recorded pairs of MLIs in voltage clamp and estimated the level of synchrony using the normalized cross-correlogram of their IPSC trains (Figure 7A; Supplemental Experimental Procedures). We found no difference in the level of synchrony between pairs of neurons sharing an electrical connection and those that did not (t test, p = 0.95, n = 36 and 50, respectively; Figure 7B). However, we found a significantly higher level of synchrony between pairs that were connected by a chemical synapse (t test, p = 0.00054, n = 18 and 68, respectively; Figure 7B). This result provides independent confirmation of the presence of transitive patterns (10, 14) in the chemical network.

### Feedforward Motifs and Their Spatial Organization

**Figure 8:**
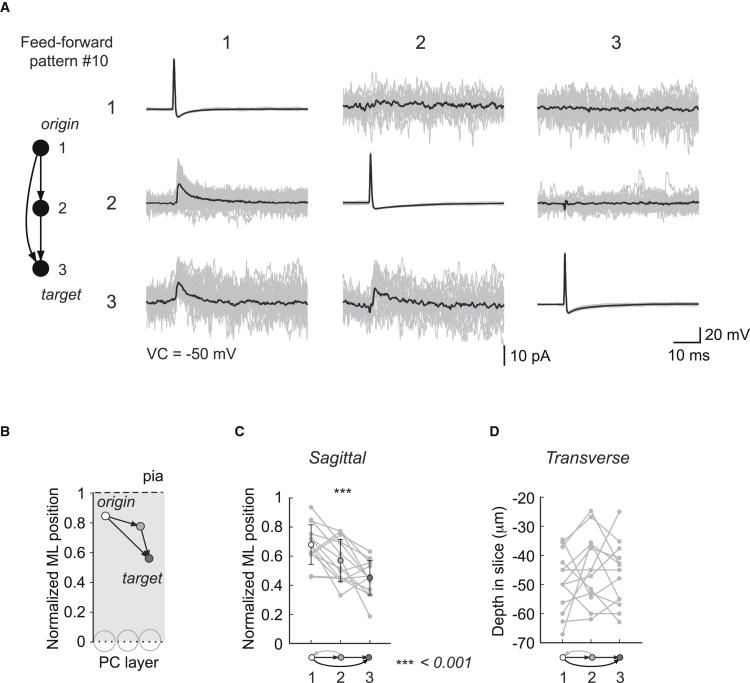
Transitive Motifs in Chemical Networks Are Oriented in the Sagittal Plane (A) Triplet recording from MLIs forming a feedforward motif. APs are elicited successively in each MLI, and the IPSCs recorded in voltage clamp (VC = −50 mV). Traces shown are averages of more than 50 sweeps. (B) Schematic showing the normalized positions of the neurons forming the feedforward pattern in the ML: the origin neuron (1) tends to be higher in the ML than the intermediate neuron (2) and the target neuron (3). (C) The positions of the neurons forming the transitive patterns (feedforward pattern 10 and regulating mutual pattern 14, n = 14) are significantly different (one-way ANOVA). (D) Positions of the neurons forming the transitive patterns along the transverse axis (n = 14, absolute depth recorded in the slice).

Although transitive connections are a signature of the chemical network (Figures 5B and 7B), it appears that the feedforward pattern (10), in particular, is a preferred motif of this network (Figures 5A and 8A; n = 13 cases). It is characterized by an origin neuron (1) sending two diverging connections, an intermediate neuron (2), and a target neuron receiving two converging connections (3). The transitivity of the feedforward (FF) motifs appeared to follow a top-to-bottom orientation in the ML, with the origin neuron being closer to the pia. To quantify this, the position in the ML of each recorded MLI was measured and normalized relative to the PC layer and pial surface (Figure 8B; Supplemental Experimental Procedures; Figure S7A). We observed that the positions of the origin and the intermediate neurons are located significantly higher in the ML than the target neuron (paired t test, p = 0.0008, p = 0.026, respectively, n = 11). Moreover, this “top-to-bottom” arrangement applies to transitive patterns, as the ML positions of their three neurons have different means (one-way ANOVA, p = 0.0002, n = 14; Figure 8C; Supplemental Information). In contrast, we found no directionality along the transverse axis: the absolute depth in the slice of the three neurons shows that individual triplets were either confined to a sagittal plane or distributed across sagittal planes without a consistent sequence (Figure 8D).

These results suggest that the position in the ML plays an important role in determining the connectivity of MLIs. Although classically MLIs have been divided into basket and stellate cells, our data support the accumulating evidence suggesting that these cells constitute a single population with a continuum of morphological properties with their position in the ML as main parameter: their dendrite length becomes gradually shorter the higher the interneuron is located in the ML (Figures S8A and S8B; Rakic, 1972; Sultan and Bower, 1998). The main axon generally maintains the same vertical position in the ML, whereas short collaterals run perpendicularly along the transverse and sagittal planes (Figures S7C and S7D). Together, these morphological arrangements explain the preference for chemical connections projecting downward in the ML (Figures S7B, S7D, and S8E) and may contribute to the high occurrence of feedforward patterns (10) and absence of loop patterns (11). We found that the underrepresentation of intransitive patterns can be well predicted by a nonuniform random model including the ML position information (Figures S5D and S5E). However, the overrepresentation of transitive patterns remained beyond what can be accounted for with ML position.

In summary, both the electrical and chemical networks display clustered and structured features of connectivity. In both networks this higher-order connectivity exhibits a specific spatial arrangement. This highlights how the functional connectivity of the interneuron network results from an interplay between the architecture of the ML and the specific connectivity motifs we have identified.

## Discussion

Using multiple whole-cell patch-clamp recordings in cerebellar slices, we provide evidence for structured features of electrical and chemical connectivity between interneurons in the cerebellar molecular layer. Although the connectivity appears mostly random at the pair level, we reveal nonrandom features of higher-order connectivity for both electrical and chemical networks. For the electrical network, we demonstrate higher-than-predicted electrical clustering and anticlustering coefficients of triplet and quadruplet patterns, supported by the confinement of electrical connections within the sagittal plane. For the chemical network, we show that transitive chemical connectivity motifs are overrepresented, with feedforward (FF) motifs being supported by a specific spatial arrangement along the sagittal plane. Finally, we find that the electrical and chemical networks are not independent at the pair and the triplet level. Together, these results indicate that the connectivity of the interneuron network is highly organized, which has important implications for the structure of activity patterns in the network.

### Evidence for Structured Connectivity in the Interneuron Network

The first evidence that neural networks are different from random networks—and exhibit small-world properties—was provided by Watts and Strogatz (1998) who used the clustering coefficient to quantify network topology. High clustering coefficients have been reported in the brain of *C. elegans* (Varshney et al., 2011; Watts and Strogatz, 1998) and extrapolated for the cortical pyramidal cell network (Perin et al., 2011). Our results provide evidence for higher-than-expected clustering in a network of only interneurons, for both electrical and chemical connectivity.

The high degree of clustering in the electrical patterns compared to random connectivity models provides strong evidence that gap junction networks exhibit clustered features in the vertebrate nervous system, as they do in *C. elegans* (Varshney et al., 2011). Although electrical connections are widespread in the mammalian brain (Bartos et al., 2002; Galarreta and Hestrin, 1999; Gibson et al., 1999; Koós and Tepper, 1999; Landisman et al., 2002; Venance et al., 2000; for review, see Connors and Long, 2004), the presence of clustered motifs in a single cell type has not previously been tested directly. Nevertheless, the dense interconnectivity mediated by gap junctions (Fukuda, 2009), the spatial organization of electrical coupling (Alcami and Marty, 2013; Amitai et al., 2002), and the segregation by cell type observed for interneurons in the cortex, striatum, and cerebellum (Blatow et al., 2003; Gibson et al., 1999; Hull and Regehr, 2012; Koós and Tepper, 1999) suggest that clustered electrical connectivity may be a general feature of interneuron networks in the mammalian brain.

We demonstrate that the interneuron chemical network also exhibits higher-than-expected clustering, as well as a preference for transitive triplet motifs. The notion of transitivity is commonly used in graph theory (Bang-Jensen and Gutin, 2008), and various complex networks have been proposed to favor locally transitive patterns, such as social networks and the World Wide Web (Holland and Leinhardt, 1970; Milo et al., 2002, 2004). We show that when examining connected triplets, interneuron networks favor motifs exhibiting transitivity, such as feedforward motifs. Previous studies of connectivity in other neural circuits have also demonstrated the overrepresentation of the feedforward motif (Jarrell et al., 2012; Kampa et al., 2006; Milo et al., 2002; Perin et al., 2011; Varshney et al., 2011) and the underrepresentation of the loop motif (Milo et al., 2002; Varshney et al., 2011). Although transitivity was not specifically investigated in these networks, it would be an interesting aspect to test, particularly given that transitivity of cortical connectivity has previously been suggested based on sequential activity of cortical neurons shown by analysis of spike time delays (Nikolić, 2007).

By simultaneously measuring both chemical and electrical connectivity in the same neurons, we show that the chemical and electrical networks established by MLIs overlap. Moreover, by analyzing higher-order connectivity, we show these two networks have a structured overlap. Strong overlap between electrical and chemical networks has been found in the *C. elegans* connectome (Varshney et al., 2011), specifically for GABAergic neurons. In mammalian interneuron networks, pairs of neurons can be connected by electrical, chemical, or both types of synapses (Blatow et al., 2003; Galarreta and Hestrin, 2002; Gibson et al., 1999; Koós and Tepper, 1999; Tamás et al., 2000). This specific overlap of both types of synapses is cell type dependent, but there is as yet no experimental evidence for a structured overlap among the same cell type. The structured overlap between the electrical and chemical networks we have observed suggests that the interactions between the two types of connections may have important roles for the function of the network.

Our results highlight the importance of probing more than two neurons in the network in order to investigate network connectivity. We observed connection specificity beyond random connectivity models and structured overlap between electrical and chemical networks at the triplet level, but only weak signs at the pair level. Different types of structured network architecture can have opposite consequences for pair connectivity. For instance, a network with a high clustering coefficient may deliver an excess of bidirectional connections, as for the network of layer 5 pyramidal cells in neocortex (Markram et al., 1997; Song et al., 2005). On the other hand, a network containing directed connectivity can result in the underrepresentation of bidirectional connections, as between excitatory cells of different cortical layers in barrel cortex (Lefort et al., 2009), and the extreme case of synaptic chains may result in the complete absence of bidirectional connections (Seung, 2009; Watt et al., 2009). Here, we find an intermediate situation, where bidirectional connections are neither overrepresented nor underrepresented despite clear signs of structured network architecture.

### What Are the Connectivity Rules?

Structured connectivity, deviating from random connectivity predictions, can result from various factors. First, deviations from random statistics may be implemented in practice by spatial constraints, such as cell morphology. In the context of the cerebellar circuit, the organization of the molecular layer along sagittal planes characterized by parallel stacks of Purkinje cell dendrites constitutes an important constraint on connectivity. The confinement of electrical coupling to the sagittal plane (Figure 2B) appears to be a consequence of this organization combined with the planar morphology of MLIs (Palay and Chan-Palay, 1974). Similarly, the gradual change in MLI morphology along the vertical axis in the molecular layer (Sultan and Bower, 1998; Figure S8) influences MLI connectivity and appears to underlie the underrepresentation of loop motifs (Figure S7D).

Second, developmental mechanisms are known to be strong determinants of neural connectivity and general network topology (Feldt et al., 2011). Aspects of connectivity may be hard-wired, genetically specified, or controlled by gradients of specific signaling molecules (Kolodkin and Tessier-Lavigne, 2011; Williams et al., 2010). Some of the connectivity motifs defined during development can play an important role in ensuring the appropriate subsequent wiring of the circuit in the cerebellum (van Welie et al., 2011).

Finally, experience and activity-dependent plasticity mechanisms have long been thought to be critical in shaping neural network architecture. Spike-timing-dependent plasticity (STDP), in particular, has been proposed to lead to structured connectivity. Modeling and theoretical studies argue that common STDP rules give rise to and maintain feedforward motifs and structures, while eliminating loops (Kozloski and Cecchi, 2010; Masuda and Kori, 2007; Ren et al., 2010; Song and Abbott, 2001; Takahashi et al., 2009). Incidentally, the increased occurrence of triplet motifs in *C. elegans*, which according to our nomenclature are transitive, can be robustly obtained from an STDP-driven network (Ren et al., 2010).

### Functional Implications

Structured connectivity can influence network dynamics and encourage correlated activity between individual neurons (Hu et al., 2012; Pernice et al., 2011; Trousdale et al., 2012). The effect of connectivity on the temporal structure of population activity is particularly interesting for interneuron networks, which can exhibit synchronization and generate oscillations (Bartos et al., 2007; Whittington and Traub, 2003). Both electrical (Draguhn et al., 1998) and inhibitory synapses (Wang and Buzsáki, 1996) can promote synchrony, and when they are combined within the same network (Fukuda and Kosaka, 2000; Galarreta and Hestrin, 2002; Koós and Tepper, 1999) they can have complementary roles and enhance synchrony (Kopell and Ermentrout, 2004; Pfeuty et al., 2007; Traub et al., 2001). However, the conditions required for this interaction are known to be dependent on various parameters, such as relative coupling strength (Kopell and Ermentrout, 2004), as well as connectivity and network topology (Buzsáki et al., 2004). Most models of synchrony are indeed based on random connectivity (Pfeuty et al., 2007; Wang and Buzsáki, 1996). In contrast, recent work has highlighted the emergence of highly spatially heterogeneous activity states when local clustering of electrical and chemical synapses is considered (Lau et al., 2010). The enhanced clustering of both electrical and chemical synaptic connections among MLIs, as well as their structured overlap, may therefore form the substrate for complex spatial patterns of network activity underlying computations in the cerebellar cortex.

A complementary way to examine the effect of different network topologies on network function is to study how different network motifs change network dynamics. Zhao et al. (2011) showed that deviations from random networks caused by overrepresenting different network motifs involving two connections in either a divergent, convergent, or chain configuration can have opposing effects on synchrony. What could be the functional consequences of the overrepresentation of transitive chemical motifs we find among MLIs? The “synaptic chain model” is an example of such a transitive network architecture containing feedforward motifs and is known to generate highly structured temporal dynamics (Abeles, 1991; Seung, 2009). Loops, on the other hand, are examples of intransitive network motifs and can generate oscillations and self-maintaining rhythms (Wang and Rinzel, 1992). Although some circuits may exploit such dynamics (Manor et al., 1999; Wang and Rinzel, 1992), the reverberating effects of loops between brain regions have been proposed to cause instability (Crick and Koch, 1998); this may also occur at the local circuit level where oscillations may lead to tremor. Thus, structured connectivity containing feedforward motifs may be beneficial for network stability. In signal processing, finite impulse response filters implemented by a feedforward motif are more stable and reliable than infinite impulse response filters implemented by a loop motif (Rabiner and Gold, 1975). It remains to be determined if such features are also exhibited by neural networks with transitive connectivity.

In the cerebellum, synchrony between MLIs (Mann-Metzer and Yarom, 1999) may be restricted to sagittal bands where electrical clustering is high. There, electrical coupling allows improved spatial averaging of the activity levels in the input population (Alcami and Marty, 2013), by sampling from a large number of parallel fibers. Inhibitory connections across sagittal planes may help synchronize successive planes with each other. Furthermore, the transitive inhibitory connectivity oriented from top to bottom of the ML may generate waves of activity traveling in the opposite direction, from the bottom to the top of the ML, by analogy to the waves along the Purkinje cell layer in the developing cerebellum (Watt et al., 2009). In summary, our quantification of the functional organization of the interneuron network places important constraints on the construction of any network model of the cerebellum (Bower, 2010; Gleeson et al., 2007; Maex and De Schutter, 2005) and should inspire many future experiments exploring the consequences of this structured connectivity for cerebellar cortical function.

## Experimental Procedures

All experiments were carried out in accordance with the animal care and handling guidelines approved by the UK Home Office. Sagittal slices of cerebellar cortex were obtained from 18- to 23-day-old rats. Slices were placed in a recording chamber perfused with standard artificial cerebrospinal fluid that contained 125 mM NaCl, 2.5 mM KCl, 2 mM CaCl_2_, 1 mM MgCl_2_, 25 mM NaHCO_3_, 1.25 mM NaH_2_PO_4_, and 25 mM D-glucose and was bubbled with carbogen (95% oxygen, 5% carbon dioxide), giving a pH of 7.4. Neurons were visualized with an upright microscope (Zeiss Axioskop) using infrared differential interference contrast (DIC) optics, optimized as described previously (Davie et al., 2006). Interneurons were identified by their soma size (10–12 μm) and their location in the molecular layer. Simultaneous whole-cell patch-clamp recordings were made at 32°C ± 1°C from up to four MLIs distributed throughout the vertical extent of the ML (Figure S8). Glass pipettes (7–10 MΩ) were filled with intracellular solution containing 130 mM K-methanesulfonate, 10 mM HEPES, 7 mM KCl, 0.05 mM EGTA, 2 mM Na_2_ATP, 2 mM MgATP, and 0.5 mM Na_2_GTP, titrated with KOH to pH 7.2. The resulting reversal potential for chloride was E_Cl^−^_ = –77.5 mV. Biocytin (0.5%) was added to the intracellular solution to label the cells. Recordings were typically made at least 30–40 μm below the surface of the slice to minimize the number of cut axons (Figure S2A). The relative position of each recorded cell in the ML was identified using the DIC image, and the intersomatic distances were read out using the stage position. MLI morphologies were reconstructed using the TREES toolbox in MATLAB (Cuntz et al., 2011), after histochemical labeling and confocal microscopy. For further details, see the Supplemental Experimental Procedures.

Data analysis was performed using Igor Pro (Wavemetrics), MATLAB (MathWorks), and Python. The probability of an electrical (p_E_) or chemical (p_C_) connection is defined as the ratio between the total number of observed connections and the total number of possible connections. For each experimentally measured pair, there is one possible electrical connection and two possible chemical connections, therefore:

$$p_{E}=n_{E}/n_{\mathrm{pairs}}$$

$$p_{C}=n_{C}/\left(2^{*}n_{\mathrm{pairs}}\right)$$

where n_E_ is the total number of electrical connections, n_C_ is the total number of chemical connections, and n_pairs_ is the total number of pairs tested. To count the occurrence of triplet patterns, all quadruplets were divided into four triplets. All triplet graphs were tested for isomorphisms for each connection type individually.

Data are reported as mean ± SD. The significance of differences between the connectivity found in the experiment and models of random connectivity was assessed using Monte Carlo methods. The first model represents the simplest case: connections between neurons are formed independently of each other based on the connection probabilities p_E_ and p_C_, and independent of other parameters. This model is called the “uniform random” model, because the probabilities p_E_ and p_C_ are uniform with respect to distance. The second model is called the “nonuniform random” model, because the probabilities of electrical and chemical connections are distance dependent and determined by the experimentally measured distribution of p_E_ and p_C_ versus the intersomatic distance between recorded cells (Figures 2A and 2B). Where appropriate, the p values were corrected for multiple hypothesis comparisons using the Bonferroni method. Further details are available in the Supplemental Experimental Procedures.

## Supplemental Information

Document S1. Supplemental Experimental Procedures and Figures S1–S8

## References

[bib1] Abeles M. (1991). Corticonics: Neural Circuits of the Cerebral Cortex.

[bib2] Alcami P., Marty A. (2013). Estimating functional connectivity in an electrically coupled interneuron network. Proc. Natl. Acad. Sci. USA.

[bib3] Amitai Y., Gibson J.R., Beierlein M., Patrick S.L., Ho A.M., Connors B.W., Golomb D. (2002). The spatial dimensions of electrically coupled networks of interneurons in the neocortex. J. Neurosci..

[bib4] Bang-Jensen J., Gutin G. (2008). Digraphs: Theory, Algorithms and Applications.

[bib5] Bartos M., Vida I., Frotscher M., Meyer A., Monyer H., Geiger J.R.P., Jonas P. (2002). Fast synaptic inhibition promotes synchronized gamma oscillations in hippocampal interneuron networks. Proc. Natl. Acad. Sci. USA.

[bib6] Bartos M., Vida I., Jonas P. (2007). Synaptic mechanisms of synchronized gamma oscillations in inhibitory interneuron networks. Nat. Rev. Neurosci..

[bib7] Blatow M., Rozov A., Katona I., Hormuzdi S.G., Meyer A.H., Whittington M.A., Caputi A., Monyer H. (2003). A novel network of multipolar bursting interneurons generates theta frequency oscillations in neocortex. Neuron.

[bib8] Bock D.D., Lee W.-C.A., Kerlin A.M., Andermann M.L., Hood G., Wetzel A.W., Yurgenson S., Soucy E.R., Kim H.S., Reid R.C. (2011). Network anatomy and in vivo physiology of visual cortical neurons. Nature.

[bib9] Bonifazi P., Goldin M., Picardo M.A., Jorquera I., Cattani A., Bianconi G., Represa A., Ben-Ari Y., Cossart R. (2009). GABAergic hub neurons orchestrate synchrony in developing hippocampal networks. Science.

[bib10] Bower J.M. (2010). Model-founded explorations of the roles of molecular layer inhibition in regulating purkinje cell responses in cerebellar cortex: more trouble for the beam hypothesis. Front. Cell Neurosci..

[bib11] Braitenberg V., Schüz A. (1991). Anatomy of the Cortex: Statistics and Geometry.

[bib12] Briggman K.L., Helmstaedter M., Denk W. (2011). Wiring specificity in the direction-selectivity circuit of the retina. Nature.

[bib13] Buzsáki G., Geisler C., Henze D.A., Wang X.-J. (2004). Interneuron Diversity series: Circuit complexity and axon wiring economy of cortical interneurons. Trends Neurosci..

[bib14] Connors B.W., Long M.A. (2004). Electrical synapses in the mammalian brain. Annu. Rev. Neurosci..

[bib15] Crick F., Koch C. (1998). Constraints on cortical and thalamic projections: the no-strong-loops hypothesis. Nature.

[bib16] Cuntz H., Forstner F., Borst A., Häusser M. (2011). The TREES toolbox—probing the basis of axonal and dendritic branching. Neuroinformatics.

[bib17] Davie J.T., Kole M.H.P., Letzkus J.J., Rancz E.A., Spruston N., Stuart G.J., Häusser M. (2006). Dendritic patch-clamp recording. Nat. Protoc..

[bib18] Denk W., Briggman K.L., Helmstaedter M. (2012). Structural neurobiology: missing link to a mechanistic understanding of neural computation. Nat. Rev. Neurosci..

[bib19] Draguhn A., Traub R.D., Schmitz D., Jefferys J.G. (1998). Electrical coupling underlies high-frequency oscillations in the hippocampus in vitro. Nature.

[bib20] Feldt S., Bonifazi P., Cossart R. (2011). Dissecting functional connectivity of neuronal microcircuits: experimental and theoretical insights. Trends Neurosci..

[bib21] Fino E., Yuste R. (2011). Dense inhibitory connectivity in neocortex. Neuron.

[bib22] Fukuda T. (2009). Network architecture of gap junction-coupled neuronal linkage in the striatum. J. Neurosci..

[bib23] Fukuda T., Kosaka T. (2000). Gap junctions linking the dendritic network of GABAergic interneurons in the hippocampus. J. Neurosci..

[bib24] Galarreta M., Hestrin S. (1999). A network of fast-spiking cells in the neocortex connected by electrical synapses. Nature.

[bib25] Galarreta M., Hestrin S. (2002). Electrical and chemical synapses among parvalbumin fast-spiking GABAergic interneurons in adult mouse neocortex. Proc. Natl. Acad. Sci. USA.

[bib26] Gibson J.R., Beierlein M., Connors B.W. (1999). Two networks of electrically coupled inhibitory neurons in neocortex. Nature.

[bib27] Gleeson P., Steuber V., Silver R.A. (2007). neuroConstruct: a tool for modeling networks of neurons in 3D space. Neuron.

[bib28] Häusser M., Clark B.A. (1997). Tonic synaptic inhibition modulates neuronal output pattern and spatiotemporal synaptic integration. Neuron.

[bib29] Helmstaedter M., Briggman K.L., Turaga S.C., Jain V., Seung H.S., Denk W. (2013). Connectomic reconstruction of the inner plexiform layer in the mouse retina. Nature.

[bib30] Hofer S.B., Ko H., Pichler B., Vogelstein J., Ros H., Zeng H., Lein E., Lesica N.A., Mrsic-Flogel T.D. (2011). Differential connectivity and response dynamics of excitatory and inhibitory neurons in visual cortex. Nat. Neurosci..

[bib31] Holland P., Leinhardt S. (1970). A Method for Detecting Structure in Sociometric Data. Am. J. Sociol..

[bib32] Hu Y., Trousdale J., Josić K., Shea-Brown E. (2012). Motif statistics and spike correlations in neuronal networks. BMC Neurosci..

[bib33] Hull C., Regehr W.G. (2012). Identification of an inhibitory circuit that regulates cerebellar Golgi cell activity. Neuron.

[bib34] Jarrell T.A., Wang Y., Bloniarz A.E., Brittin C.A., Xu M., Thomson J.N., Albertson D.G., Hall D.H., Emmons S.W. (2012). The connectome of a decision-making neural network. Science.

[bib35] Jiang X., Wang G., Lee A.J., Stornetta R.L., Zhu J.J. (2013). The organization of two new cortical interneuronal circuits. Nat. Neurosci..

[bib36] Jörntell H., Bengtsson F., Schonewille M., De Zeeuw C.I. (2010). Cerebellar molecular layer interneurons - computational properties and roles in learning. Trends Neurosci..

[bib37] Kampa B.M., Letzkus J.J., Stuart G.J. (2006). Cortical feed-forward networks for binding different streams of sensory information. Nat. Neurosci..

[bib38] Kätzel D., Zemelman B.V., Buetfering C., Wölfel M., Miesenböck G. (2011). The columnar and laminar organization of inhibitory connections to neocortical excitatory cells. Nat. Neurosci..

[bib39] Ko H., Hofer S.B., Pichler B., Buchanan K.A., Sjöström P.J., Mrsic-Flogel T.D. (2011). Functional specificity of local synaptic connections in neocortical networks. Nature.

[bib40] Kolodkin A.L., Tessier-Lavigne M. (2011). Mechanisms and molecules of neuronal wiring: a primer. Cold Spring Harb. Perspect. Biol..

[bib41] Kondo S., Marty A. (1998). Synaptic currents at individual connections among stellate cells in rat cerebellar slices. J. Physiol..

[bib42] Koós T., Tepper J.M. (1999). Inhibitory control of neostriatal projection neurons by GABAergic interneurons. Nat. Neurosci..

[bib43] Kopell N., Ermentrout B. (2004). Chemical and electrical synapses perform complementary roles in the synchronization of interneuronal networks. Proc. Natl. Acad. Sci. USA.

[bib44] Kozloski J., Cecchi G.A. (2010). A theory of loop formation and elimination by spike timing-dependent plasticity. Front. Neural Circuits.

[bib45] Landisman C.E., Long M.A., Beierlein M., Deans M.R., Paul D.L., Connors B.W. (2002). Electrical synapses in the thalamic reticular nucleus. J. Neurosci..

[bib46] Lau T., Gage G.J., Berke J.D., Zochowski M. (2010). Local dynamics of gap-junction-coupled interneuron networks. Phys. Biol..

[bib47] Lefort S., Tomm C., Floyd Sarria J.-C., Petersen C.C.H. (2009). The excitatory neuronal network of the C2 barrel column in mouse primary somatosensory cortex. Neuron.

[bib48] Letzkus J.J., Wolff S.B.E., Meyer E.M.M., Tovote P., Courtin J., Herry C., Lüthi A. (2011). A disinhibitory microcircuit for associative fear learning in the auditory cortex. Nature.

[bib49] Llano I., Gerschenfeld H.M. (1993). Inhibitory synaptic currents in stellate cells of rat cerebellar slices. J. Physiol..

[bib50] Maex R., De Schutter E. (2005). Oscillations in the cerebellar cortex: a prediction of their frequency bands. Prog. Brain Res..

[bib51] Maisak M.S., Haag J., Ammer G., Serbe E., Meier M., Leonhardt A., Schilling T., Bahl A., Rubin G.M., Nern A. (2013). A directional tuning map of Drosophila elementary motion detectors. Nature.

[bib52] Mann-Metzer P., Yarom Y. (1999). Electrotonic coupling interacts with intrinsic properties to generate synchronized activity in cerebellar networks of inhibitory interneurons. J. Neurosci..

[bib53] Manor Y., Nadim F., Epstein S., Ritt J., Marder E., Kopell N. (1999). Network oscillations generated by balancing graded asymmetric reciprocal inhibition in passive neurons. J. Neurosci..

[bib54] Markram H. (2006). The blue brain project. Nat. Rev. Neurosci..

[bib55] Markram H., Lübke J., Frotscher M., Roth A., Sakmann B. (1997). Physiology and anatomy of synaptic connections between thick tufted pyramidal neurones in the developing rat neocortex. J. Physiol..

[bib56] Masuda N., Kori H. (2007). Formation of feedforward networks and frequency synchrony by spike-timing-dependent plasticity. J. Comput. Neurosci..

[bib57] Milo R., Shen-Orr S., Itzkovitz S., Kashtan N., Chklovskii D., Alon U. (2002). Network motifs: simple building blocks of complex networks. Science.

[bib58] Milo R., Itzkovitz S., Kashtan N., Levitt R., Shen-Orr S., Ayzenshtat I., Sheffer M., Alon U. (2004). Superfamilies of evolved and designed networks. Science.

[bib59] Mittmann W., Koch U., Häusser M. (2005). Feed-forward inhibition shapes the spike output of cerebellar Purkinje cells. J. Physiol..

[bib60] Nikolić D. (2007). Non-parametric detection of temporal order across pairwise measurements of time delays. J. Comput. Neurosci..

[bib61] Packer A.M., Yuste R. (2011). Dense, unspecific connectivity of neocortical parvalbumin-positive interneurons: a canonical microcircuit for inhibition?. J. Neurosci..

[bib62] Palay S., Chan-Palay V. (1974). Cerebellar Cortex: Cytology and Organization.

[bib63] Perin R., Berger T.K., Markram H. (2011). A synaptic organizing principle for cortical neuronal groups. Proc. Natl. Acad. Sci. USA.

[bib64] Pernice V., Staude B., Cardanobile S., Rotter S. (2011). How structure determines correlations in neuronal networks. PLoS Comput. Biol..

[bib65] Peters A., Feldman M.L. (1976). The projection of the lateral geniculate nucleus to area 17 of the rat cerebral cortex. I. General description. J. Neurocytol..

[bib66] Pfeuty B., Golomb D., Mato G., Hansel D. (2007). Inhibition potentiates the synchronizing action of electrical synapses. Front. Comput. Neurosci..

[bib67] Pi H.-J., Hangya B., Kvitsiani D., Sanders J.I., Huang Z.J., Kepecs A. (2013). Cortical interneurons that specialize in disinhibitory control. Nature.

[bib68] Rabiner L., Gold B. (1975). Theory and Application of Digital Signal Processing.

[bib69] Rakic P. (1972). Extrinsic cytological determinants of basket and stellate cell dendritic pattern in the cerebellar molecular layer. J. Comp. Neurol..

[bib70] Ren Q., Kolwankar K.M., Samal A., Jost J. (2010). STDP-driven networks and the C. elegans neuronal network. Phys. A Stat. Mech. Its Appl..

[bib71] Seung H.S. (2009). Reading the book of memory: sparse sampling versus dense mapping of connectomes. Neuron.

[bib72] Sippy T., Yuste R. (2013). Decorrelating action of inhibition in neocortical networks. J. Neurosci..

[bib73] Song S., Abbott L.F. (2001). Cortical development and remapping through spike timing-dependent plasticity. Neuron.

[bib74] Song S., Sjöström P.J., Reigl M., Nelson S., Chklovskii D.B. (2005). Highly nonrandom features of synaptic connectivity in local cortical circuits. PLoS Biol..

[bib75] Sotelo C., Llinás R. (1972). Specialized membrane junctions between neurons in the vertebrate cerebellar cortex. J. Cell Biol..

[bib76] Sultan F., Bower J.M. (1998). Quantitative Golgi study of the rat cerebellar molecular layer interneurons using principal component analysis. J. Comp. Neurol..

[bib77] Takahashi Y.K., Kori H., Masuda N. (2009). Self-organization of feed-forward structure and entrainment in excitatory neural networks with spike-timing-dependent plasticity. Phys. Rev. E Stat. Nonlin. Soft Matter Phys..

[bib78] Takemura S.Y., Bharioke A., Lu Z., Nern A., Vitaladevuni S., Rivlin P.K., Katz W.T., Olbris D.J., Plaza S.M., Winston P. (2013). A visual motion detection circuit suggested by Drosophila connectomics. Nature.

[bib79] Tamás G., Buhl E.H., Lörincz A., Somogyi P. (2000). Proximally targeted GABAergic synapses and gap junctions synchronize cortical interneurons. Nat. Neurosci..

[bib80] Traub R.D., Kopell N., Bibbig A., Buhl E.H., LeBeau F.E., Whittington M.A. (2001). Gap junctions between interneuron dendrites can enhance synchrony of gamma oscillations in distributed networks. J. Neurosci..

[bib81] Trousdale J., Hu Y., Shea-Brown E., Josić K. (2012). Impact of network structure and cellular response on spike time correlations. PLoS Comput. Biol..

[bib82] van Welie I., Smith I.T., Watt A.J. (2011). The metamorphosis of the developing cerebellar microcircuit. Curr. Opin. Neurobiol..

[bib83] Varshney L.R., Chen B.L., Paniagua E., Hall D.H., Chklovskii D.B. (2011). Structural properties of the Caenorhabditis elegans neuronal network. PLoS Comput. Biol..

[bib84] Venance L., Rozov A., Blatow M., Burnashev N., Feldmeyer D., Monyer H. (2000). Connexin expression in electrically coupled postnatal rat brain neurons. Proc. Natl. Acad. Sci. USA.

[bib85] Vervaeke K., Lőrincz A., Gleeson P., Farinella M., Nusser Z., Silver R.A. (2010). Rapid desynchronization of an electrically coupled interneuron network with sparse excitatory synaptic input. Neuron.

[bib86] Vincent P., Marty A. (1993). Neighboring cerebellar Purkinje cells communicate via retrograde inhibition of common presynaptic interneurons. Neuron.

[bib87] Wang X.J., Buzsáki G. (1996). Gamma oscillation by synaptic inhibition in a hippocampal interneuronal network model. J. Neurosci..

[bib88] Wang X.-J., Rinzel J. (1992). Alternating and synchronous rhythms in reciprocally inhibitory model neurons. Neural Comput..

[bib89] Watt A.J., Cuntz H., Mori M., Nusser Z., Sjöström P.J., Häusser M. (2009). Traveling waves in developing cerebellar cortex mediated by asymmetrical Purkinje cell connectivity. Nat. Neurosci..

[bib90] Watts D.J., Strogatz S.H. (1998). Collective dynamics of ‘small-world’ networks. Nature.

[bib91] Whittington M.A., Traub R.D. (2003). Interneuron diversity series: inhibitory interneurons and network oscillations in vitro. Trends Neurosci..

[bib92] Williams M.E., de Wit J., Ghosh A. (2010). Molecular mechanisms of synaptic specificity in developing neural circuits. Neuron.

[bib93] Yoshimura Y., Callaway E.M. (2005). Fine-scale specificity of cortical networks depends on inhibitory cell type and connectivity. Nat. Neurosci..

[bib94] Yoshimura Y., Dantzker J.L.M., Callaway E.M. (2005). Excitatory cortical neurons form fine-scale functional networks. Nature.

[bib95] Yu S., Huang D., Singer W., Nikolic D. (2008). A small world of neuronal synchrony. Cereb. Cortex.

[bib96] Zhao L., Beverlin B., Netoff T., Nykamp D.Q. (2011). Synchronization from second order network connectivity statistics. Front. Comput. Neurosci..

