# Supplementary material for: Structured Connectivity in Cerebellar Inhibitory Networks
Source: Neuron. 2014 Feb 19;81(4):913–29. doi: 10.1016/j.neuron.2013.12.029 (PMC3988957; doi:10.1016/j.neuron.2013.12.029)
Supplement: Document S1. Supplemental Experimental Procedures and Figures S1–S8 [file mmc1.pdf]

Neuron, Volume *81*

Supplemental Information

# Structured Connectivity in Cerebellar Inhibitory Networks

Sarah Rieubland, Arnd Roth, and Michael Häusser

## Supplemental Information, Rieubland et al.

## Inventory of Supplemental Information

### *Supplemental Data:*

**Figure S1** shows examples of the postsynaptic responses for the three connection types, in both current clamp and voltage clamp, in addition to the one shown in Figure 1.

**Figure S2** shows that the measured probabilities of connection (Figure 2) are not affected by the slicing procedure. Figure S2 also shows the distribution of intersomatic distances in xy and z, as well as the full interpolated functions of $p_E$ and $p_C$, which are underlying Figure 2A,B.

**Figure S3** describes the random connectivity models with ML position, in addition to the two random models described in Figure 3A.

**Figure S4** shows the results for the random models with ML position when comparing pair connectivity (Figure 3) and electrical triplet connectivity (Figure 4).

**Figure S5** provides a full description of all 16 chemical triplet patterns in Figure 5 and shows the results for the random models with ML position when comparing chemical triplet connectivity (Figure 5).

**Figure S6** shows the results for the non-uniform random models with ML position for the common neighbor analysis (Figure 6).

**Figure S7** shows the influence of the ML position on morphology and connectivity of MLIs, as suggested by the results of Figure 8.

**Figure S8** provides evidence for the hypothesis that MLIs represent a continuous population with properties changing across the ML.

### *Supplemental Experimental Procedures:*

This includes detailed descriptions of the Experimental Procedures, additional details on the Monte Carlo methods for statistical analysis, and the use of the ML position parameter for building the two additional random connectivity models.

## Supplemental Data

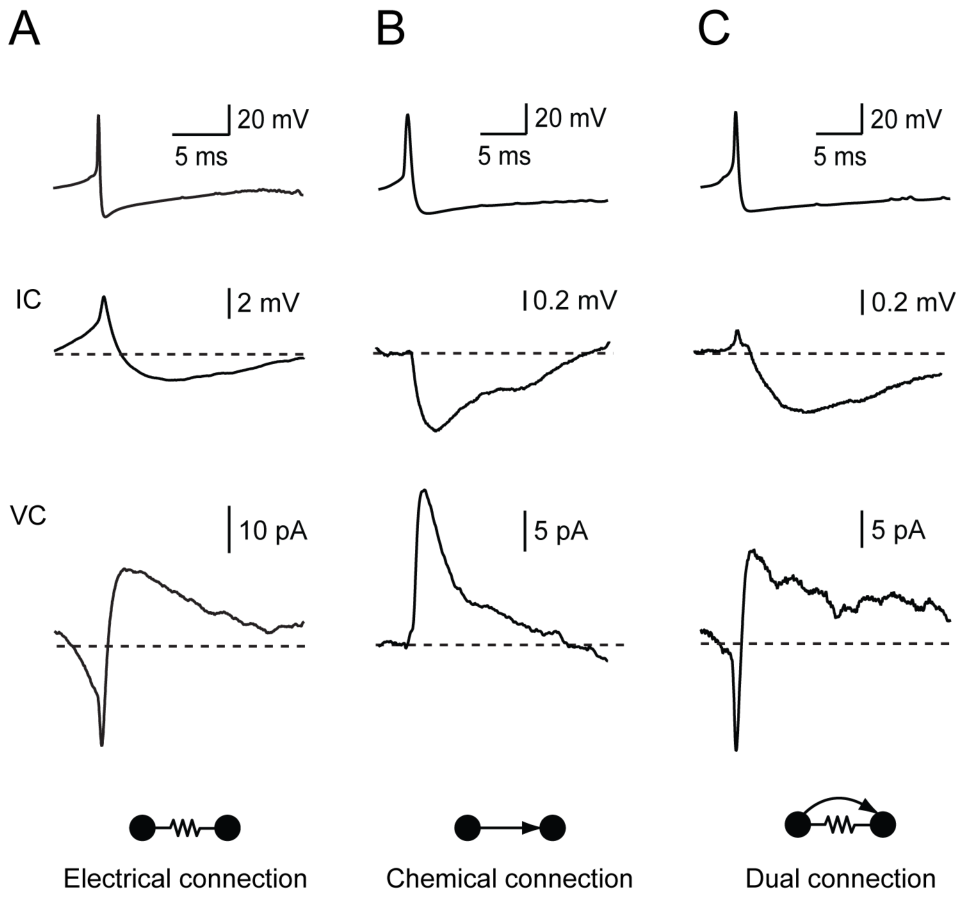


**Figure S1. Voltage and current clamp recordings of postsynaptic responses in three different connection types**

(A) Electrical connection: an action potential (AP) in the presynaptic cell triggers a post-synaptic spikelet and the transmission of the after-hyperpolarisation (AHP; average of > 20 sweeps). (B) Chemical connection: an AP triggers an IPSP in current clamp (IC) or an IPSC in voltage clamp (VC). (C) Dual connection: the postsynaptic response to a presynaptic AP is composed of a spikelet followed by a combination of the coupled AHP and the IPSP/IPSC.

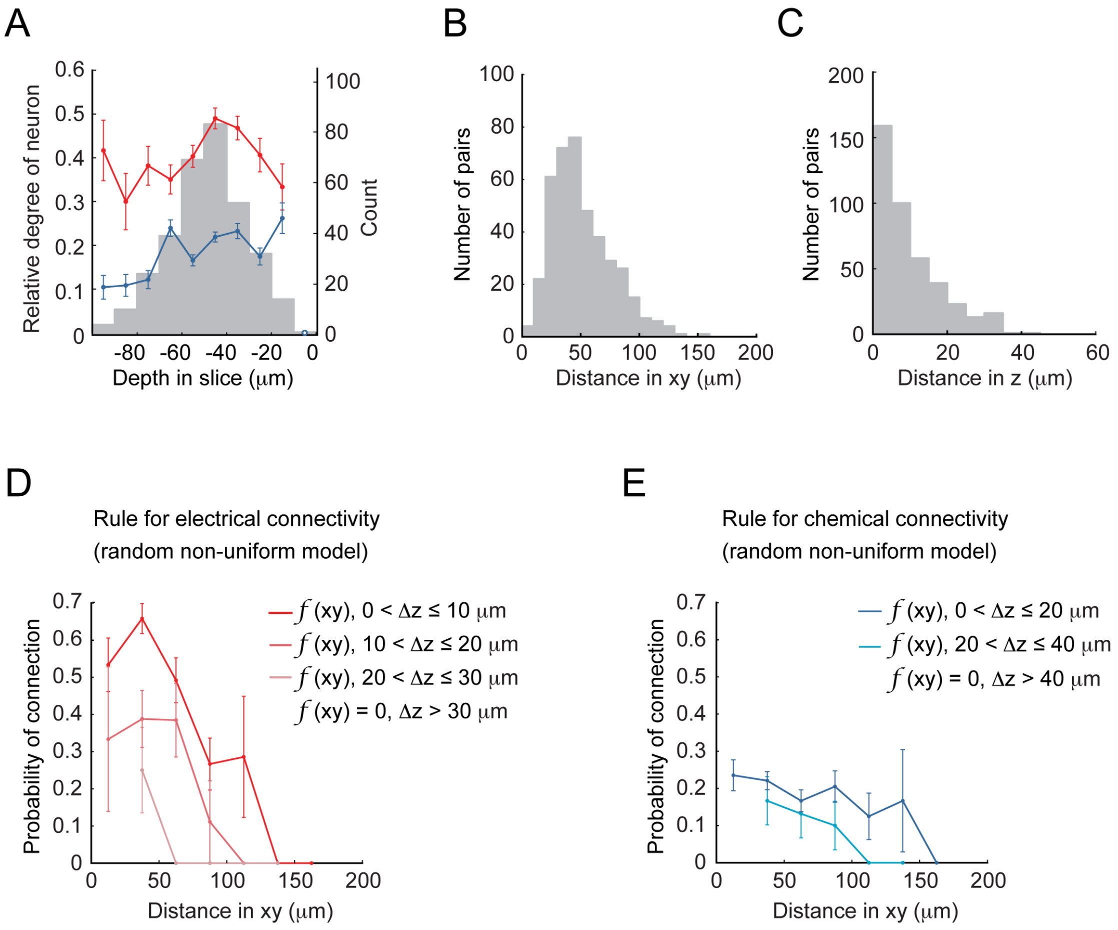


**Figure S2. Distributions of the position of recorded MLIs and model interpolation for $p_E$ and $p_C$**

(A) Histogram of absolute depth in the slice of the somata of 329 MLIs. The relative degree (number of connections / number of possible connections) of the MLI for electrical (red) and chemical (blue) connectivity is shown superimposed. (B,C) Distribution of intersomatic distances between recorded MLI pairs, in the sagittal plane (xy; B) and along the transverse axis (z; C). (D) Probability of electrical connections vs. distance between pairs in xy, for 3 different intervals of intersomatic distances in z ($p_E$ = 0 for $\Delta z$ > 40 μm). The 3 resulting interpolation functions $f$(xy) are used for the prediction of the non-uniform random model. (E) Probability of chemical connections vs. distance in xy for two different intervals of intersomatic distances in z ($p_C$ = 0 for $\Delta z$ > 40 μm). The resulting interpolation functions $f$(xy) are used for the prediction of the non-uniform random model.

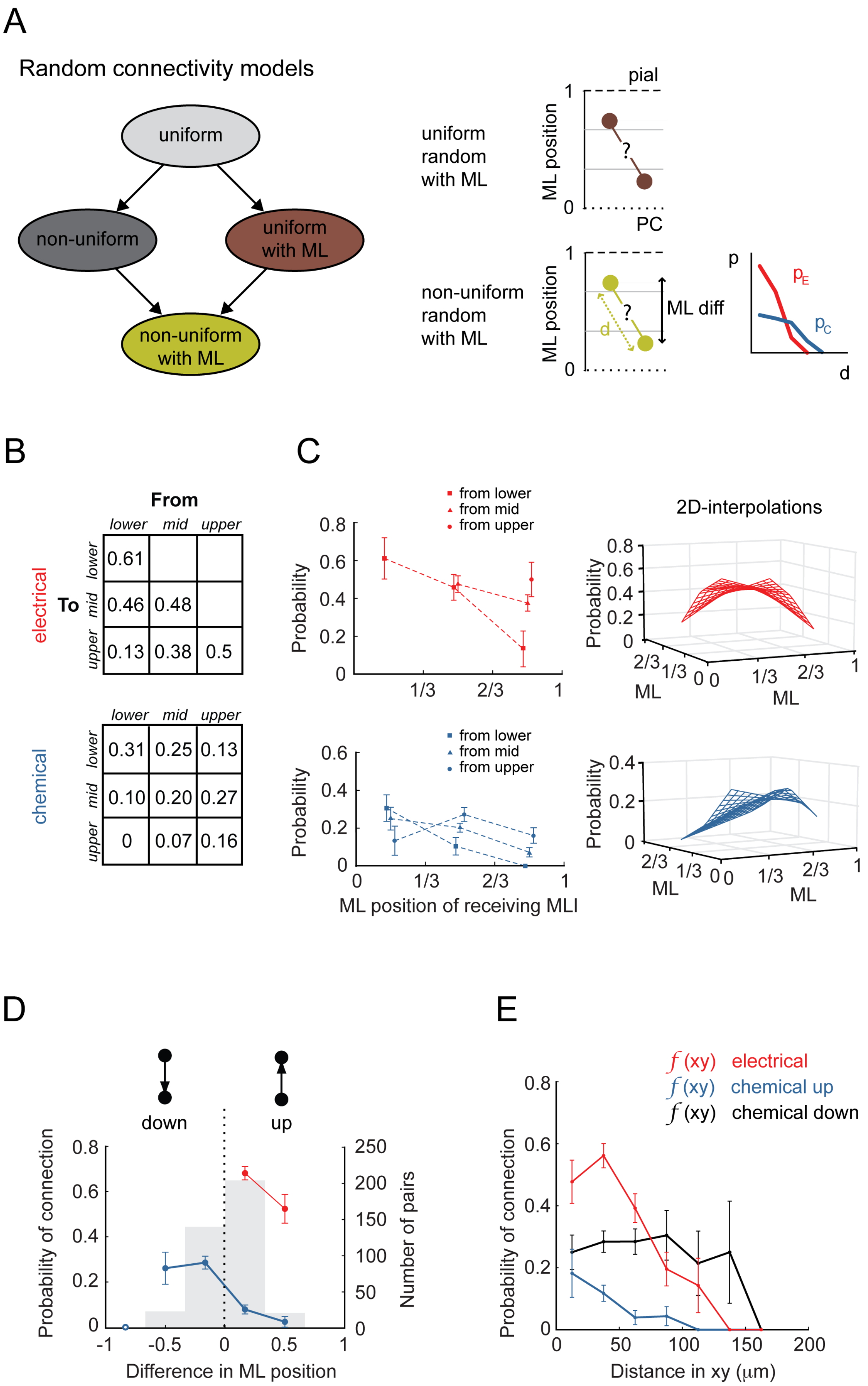

A
Random connectivity models
uniform
non-uniform
uniform with ML
non-uniform with ML
uniform random with ML
non-uniform random with ML
pial
PC
ML position
ML diff
d
p
$p_E$
$p_C$
B
C
From
To
lower mid upper
electrical
0.61
0.46 0.48
0.13 0.38 0.5
chemical
0.31 0.25 0.13
0.10 0.20 0.27
0 0.07 0.16
from lower
from mid
from upper
Probability
2D-interpolations
ML
ML position of receiving MLI
D
down
up
Probability of connection
Number of pairs
Difference in ML position
E
f (xy) electrical
f (xy) chemical up
f (xy) chemical down
Distance in xy (μm)

**Figure S3. Random connectivity models including molecular layer (ML) position**

(A) Schematic illustrating the hierarchy of random models. The simplest assumption is the uniform random model. Including parameters influencing connectivity, such as intersomatic distance (non-uniform random model) or position in the ML (uniform random with ML position model) is a way to test the predictive power of these parameters on connectivity. The non-uniform random with ML position model combines both parameters. These models remain probabilistic despite being constrained by these parameters. (B) Probability of electrical (top) and chemical (bottom) connection matrix between the 3 thirds (lower, mid, upper) of the ML. Electrical connections count as bidirectional, therefore only one side of the matrix is filled. (C) Graphical representation of the probability of connection matrices in B, as a function of the ML position of the receiving MLI (left). Traces are slightly offset in the x axis for clarity. The matrices can be represented in a plane and interpolated as 2D functions (right). (D) Histogram of the differences of ML position for the recorded pairs and probabilities of electrical and chemical connections vs. difference in ML position. Positive ML position differences are for chemical connections going up and negative ML position differences are for chemical connections going down. (E) Linear interpolation functions for the probability of electrical, chemical up and chemical down connections, as a function of intersomatic distances in xy.

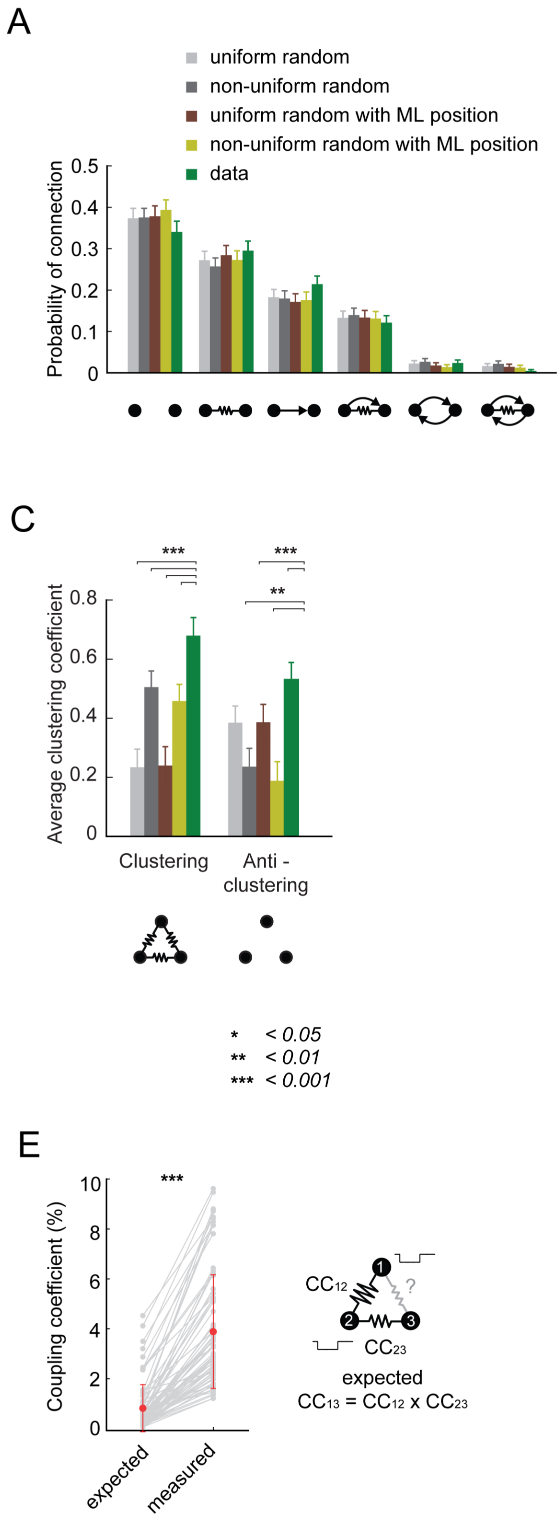


**Figure S4. Predictions of all four random connectivity models for pair connectivity and electrical higher order connectivity**

(A) Probability of occurrence of the 6 possible connection types between pairs, in the data and predicted by the four random connectivity models. (B) Relative occurrence of each connection type in the data compared to the

prediction of the four random models. (C) Average clustering and anti-clustering coefficient of the triplet and quadruplet electrical patterns, in the data and predicted by the four random models. (D) Probability of occurrence of the four possible electrical triplet patterns in the data and predicted by the four random models (top). Relative occurrences of each pattern in the data compared to the prediction of the four random models (bottom). (E) All electrical connections in fully connected triplets are direct connections. The coupling coefficient (CC) of the fully connected triplets was tested for contributions due to indirect coupling. The expected value of CC of the smallest connection, $CC_{13}$, was estimated based on the two largest CC connections as $CC_{13} = CC_{12} \times CC_{23}$. The measured value of $CC_{13}$ was higher than the one estimated assuming indirect coupling (t test, $p = 2.73 \times 10^{-25}$, $n = 76$). We conclude that the observed fully connected triplets (pattern #4) are indeed the result of direct connections and not indirect electrical coupling.

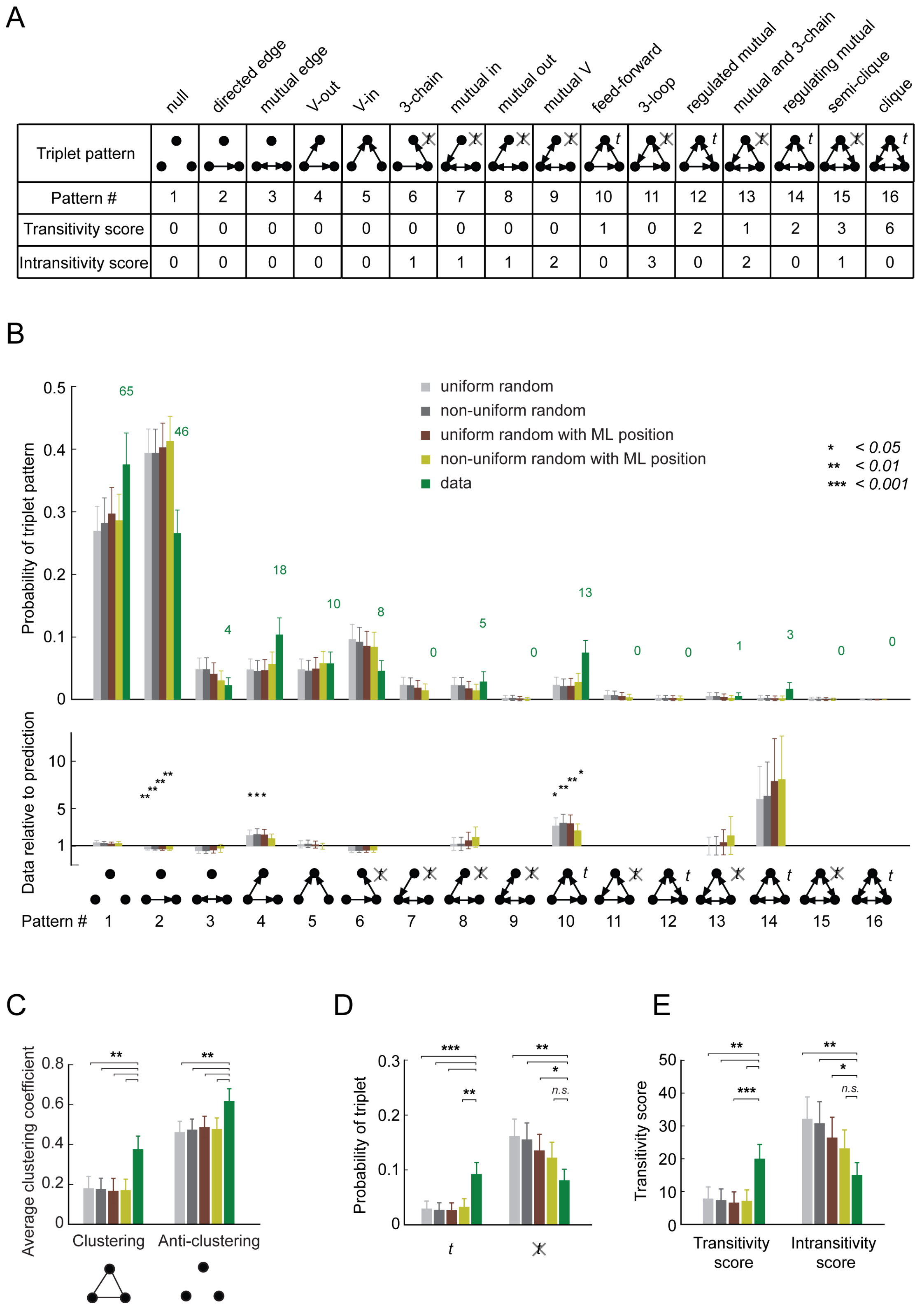


**Figure S5. Predictions of all four random connectivity models for chemical higher order connectivity**

(A) All 16 non-isomorphic chemical triplet patterns with their names (Milo et al., 2004), their transitivity score (number of transitive relations in the pattern)

and intransitivity score (number of intransitive relations in the pattern). (B) Probability of occurrence of the 16 chemical triplet patterns in the data and predicted by the four random models (top). Relative occurrence of each pattern in the data compared to the prediction of the four random models (bottom). (C) Average clustering and anti-clustering coefficient of the triplet and quadruplet chemical patterns, in the data and predicted by the four random models. (D) Probability of occurrence of transitive and intransitive triplet patterns in the data and the four random models. (E) Transitivity and intransitivity scores in the data and the four random models.

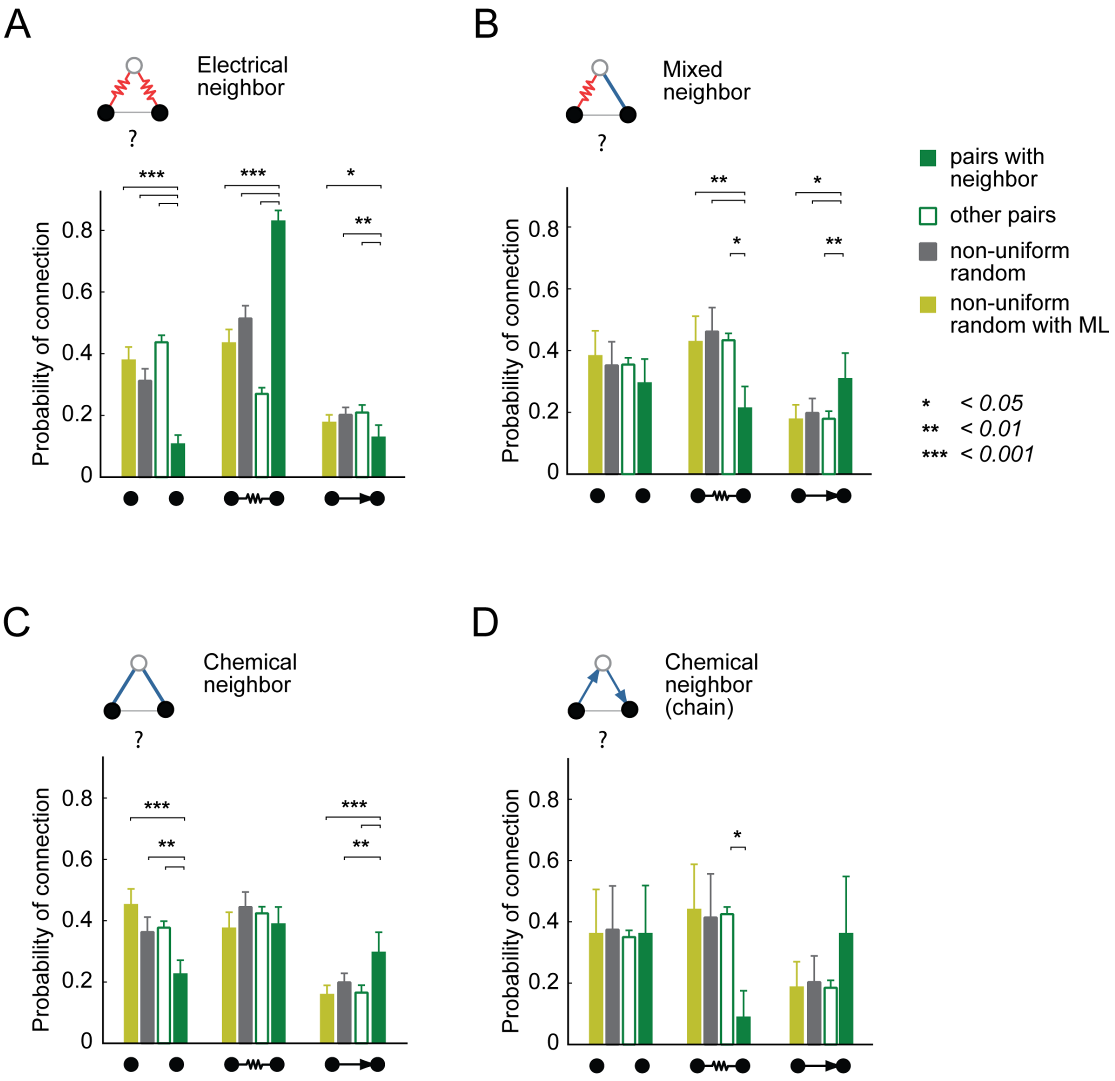


**Figure S6. Common neighbor analysis with the non-uniform random model with ML position**

(A-D) Common neighbor analysis comparing the probability of finding no connection, an electrical or a chemical connection between pairs with a common neighbor (green), with no common neighbor (white), predicted based on the intersomatic distances between the pairs with a common neighbor (non-uniform random model; gray) and predicted based on the intersomatic distances and the ML positions of the pairs with a common neighbor (non-uniform random with ML model; yellow). (A) Pairs sharing an electrical neighbor. (B) Pairs sharing a mixed (electrical only and chemical only) neighbor. (C) Pairs sharing a chemical neighbor (any direction). (D) Pairs sharing a chemical neighbor in a chain configuration.

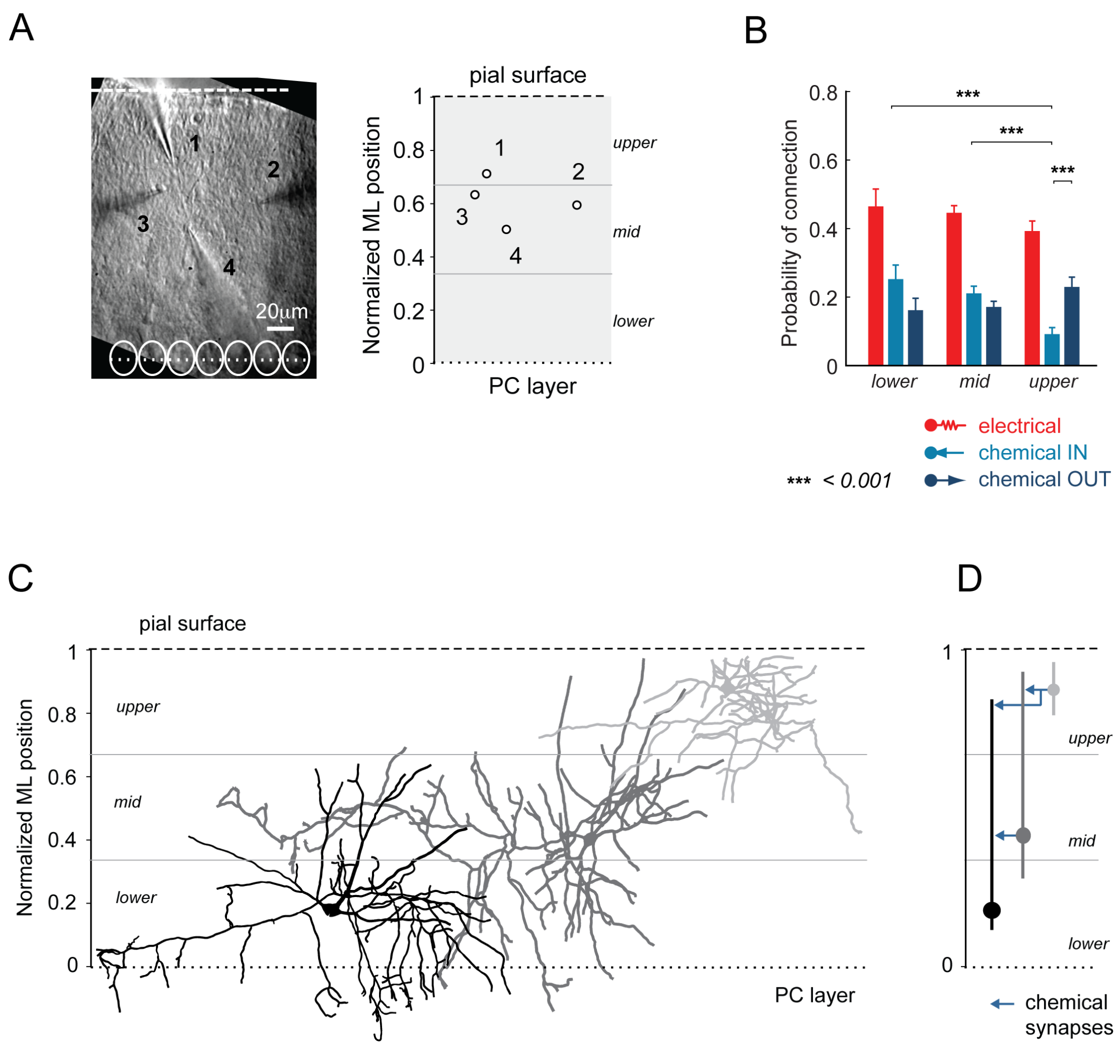


**Figure S7. The influence of ML position on MLI connectivity and morphology**

(A) The ML positions of the recorded MLIs were determined based on the DIC images and normalized using the position of the PC layer (position = 0) and the position of the pial surface (position = 1). (B) The probability of forming a connection is related to the position of the MLI in the ML. Probability of forming an electrical connection (red), receiving a chemical (light blue) and sending a chemical connection (dark blue) for each third of the ML. A $\chi^2$ test was used to test for significance of differences. (C) Example of three reconstructed MLI morphologies located in each of the "thirds" of the ML. (D). Schematic describing the preferred chemical connectivity based on the characteristic morphologies. The length of the MLI dendrites decreases with

ML position, while the main axon stays at the same level in the ML as the soma.

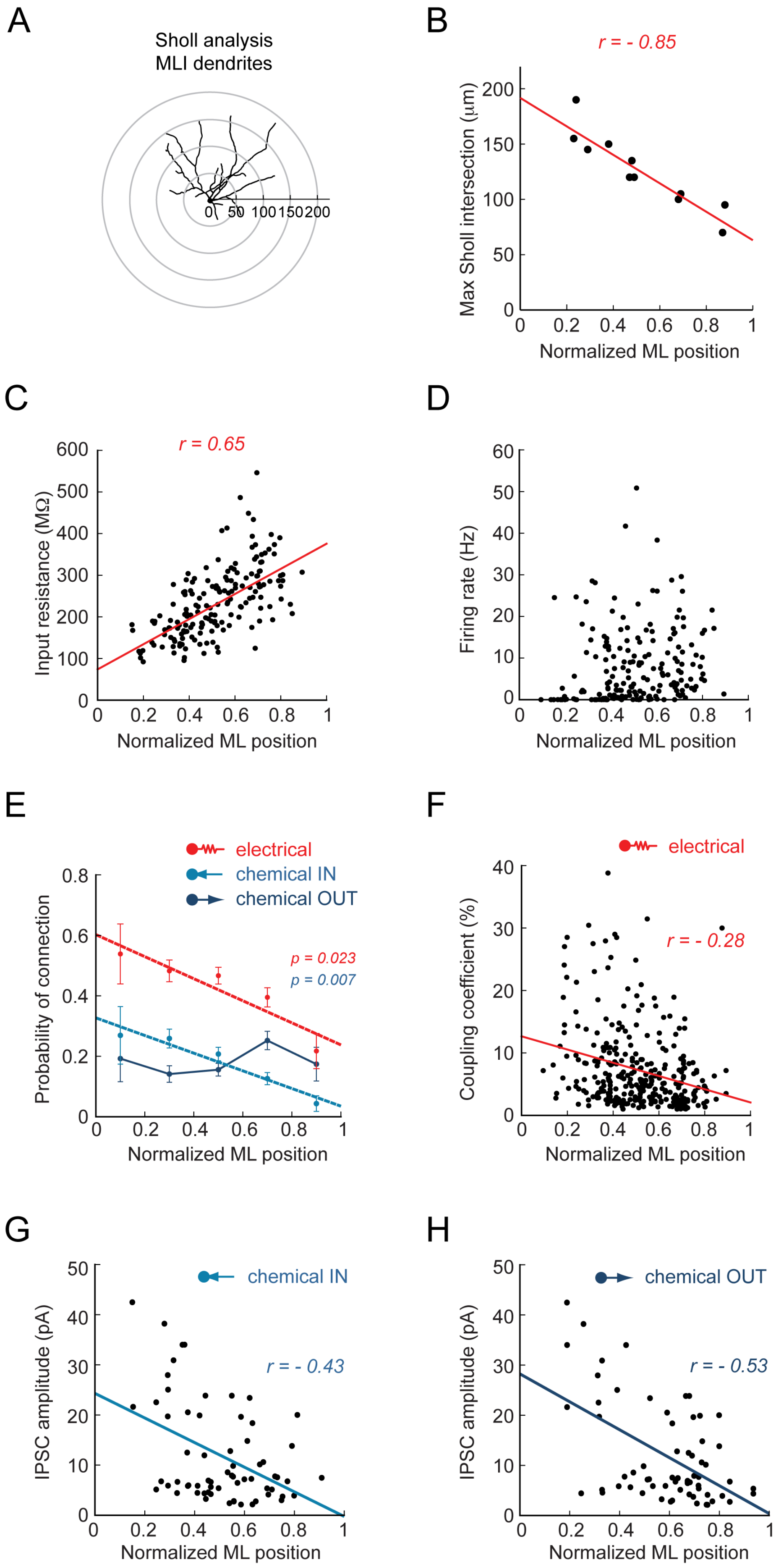
A
Sholl analysis
MLI dendrites
0
50
100
150
200
B
r = - 0.85
Max Sholl intersection (µm)
Normalized ML position
C
r = 0.65
Input resistance (MΩ)
Normalized ML position
D
Firing rate (Hz)
Normalized ML position
E
electrical
chemical IN
chemical OUT
p = 0.023
p = 0.007
Probability of connection
Normalized ML position
F
electrical
r = - 0.28
Coupling coefficient (%)
Normalized ML position
G
chemical IN
r = - 0.43
IPSC amplitude (pA)
Normalized ML position
H
chemical OUT
r = - 0.53
IPSC amplitude (pA)
Normalized ML position

**Figure S8. MLIs form a continuous population across the ML: evidence from morphology, physiology and connectivity.**

(A) Sholl analysis of the dendritic tree of 12 MLIs reconstructed from confocal microscopy (only one tree is shown here, distances in μm). (B) Maximal intersection from Sholl analysis vs. position of the MLI in the ML. Correlation coefficient r = -0.85. (C) The MLI input resistance is correlated with the position of the MLI in the ML. Correlation coefficient r = 0.65. (D) The spontaneous firing frequency of MLIs is broadly distributed across cells in the ML and does not abruptly change between lower and upper ML. (E) Probability of connection (electrical, receiving a chemical, sending a chemical) vs. position of the MLI in the ML. The probability of electrical and the probability of receiving a chemical connection continuously decreases with ML position (linear fit, p = 0.023 and p = 0.007, respectively), consistent with the change in morphology. (F) Coupling coefficient vs. ML position. There is no segregation of coupling strength across the ML (correlation coefficient r = -0.28). (G) IPSC amplitude vs ML position of the receiving MLI. (H) IPSC amplitude vs. ML position of the sending MLI. There is no strong segregation of connection strengths across the ML (correlation coefficients r = -0.43 and r = -0.53, respectively).

## Supplemental Experimental Procedures

*Slice preparation*

250 µm thick slices of cerebellar cortex were made from 18-23 day old rats using standard techniques. Briefly, rats were anaesthetized with isoflurane and decapitated in accordance with UK Home Office regulations. The brain was extracted after dissection in ice-cold artificial cerebrospinal fluid (ACSF), containing low sodium concentration and sucrose (slicing ACSF) (Clark and Cull-Candy, 2002). Slices were cut using a vibratome (Leica VT1200S), after z-axis vibration was minimized. The slices were incubated in carbogen-saturated ACSF at 34$^{o}$C for 30 min and then at room temperature for at least 30 min. During incubation the solution was slowly exchanged between slicing and standard ACSF. Standard ACSF contained (in mM) 125 NaCl, 2.5 KCl, 2 $CaCl_2$, 1 $MgCl_2$, 25 $NaHCO_3$, 1.25 $NaH_2PO_4$ and 25 D-glucose and was bubbled with carbogen (95% oxygen, 5% carbon dioxide), giving a pH of 7.4. Slicing ACSF contained (in mM) 90 NaCl, 2.5 KCl, 2 $CaCl_2$, 1 $MgCl_2$, 25 $NaHCO_3$, 1.25 $NaH_2PO_4$, 25 D-glucose and 70 sucrose.

*Multiple patch-clamp recordings*

Slices were placed in a standard ACSF-perfused recording chamber and visualized with an upright microscope (Zeiss Axioskop) using infrared-differential interference contrast (DIC) optics, optimized as described previously (Davie et al., 2006). All recordings were made at 32 ± 1 °C. Interneurons were identified by their soma size (10-12 µm) and location in the molecular layer. Simultaneous whole-cell current-clamp and voltage-clamp recordings were made from the soma of interneurons using two dual-channel amplifiers (Multiclamp 700B). Glass pipettes (7-10 MΩ) were filled with intracellular solution containing (in mM) 130 K-methanesulfonate, 10 HEPES, 7 KCl, 0.05 EGTA, 2 $Na_2ATP$, 2 MgATP and 0.5 $Na_2GTP$, titrated with KOH to pH 7.2. The resulting reversal potential for chloride is $E_{Cl-}$ = -77.5 mV. Biocytin (0.5%) was added to the intracellular solution to label the cells. Recordings were typically made at least 30 - 40 µm below the surface of the slice to minimize the number of cut axons (Figure S2A). Pair, triplet and quadruplet

simultaneous recordings were achieved by first establishing cell-attached recordings and then breaking in. Compensation for access resistance and pipette capacitance was performed and monitored throughout the recording. The recorded traces were low-pass filtered at 3 kHz, digitized at 40 kHz and acquired using Axograph software. The traces were then imported into Igor Pro (Wavemetrics) for analysis. The relative position of each recorded cell in the ML was identified using the DIC image and the intersomatic distances were read out using the stage position. Some cells were also filled with Alexa 488 or Alexa 594 and imaged under a two-photon microscope. Interneurons had an average input resistance $R_{input} = 205 \pm 102$ MΩ (n = 334; Figure S8C) and a spontaneous firing rate of 7.9 ± 8.7 Hz (n = 326; Figure S8D).

*Identification of connections*

To test for the presence of chemical and electrical synaptic connections, cells were hyperpolarized to -65 to -70 mV, and injected with short and long current pulses. Electrical coupling was evaluated by injecting steady-state current pulses in the presynaptic cell (-100 pA, 400 ms) and recording the voltage responses in both cells (averaging > 20 traces). The coupling coefficient was calculated as $CC_{12} = \Delta V_2 / \Delta V_1$, where $\Delta V_1$ and $\Delta V_2$ are the voltage differences compared to baseline in the pre- and post-junctional cell, respectively. Pairs were considered electrically coupled if the coupling coefficient satisfied CC ≥ 1% in both directions. Using an alternative criterion (the mean of the two coupling coefficients in each direction ≥ 1%) did not alter the statistics of connections and the results presented. In order to detect the presence of inhibitory connections, action potentials were elicited in the presynaptic cell at 1 Hz (1 nA, 1 ms pulses) while averaging (> 20 traces) the postsynaptic subthreshold membrane potential in current clamp mode (IC), or the postsynaptic current in voltage clamp (VC) mode at a holding potential of -50 mV. When the cell was spontaneously active, online spike-triggered averaging was performed to confirm the connection. Both IPSC and IPSP amplitudes were measured (Figure S1). Interneuron pairs with strong electrical coupling were assessed carefully, as weak chemical connections can be obstructed by the large coupled after-hyperpolarization (AHP). If the coupling coefficient of

the AHP ($CC_{AHP}$) was larger than the steady state CC, or if a faster rise time of the IPSP/C and a larger variance of the IPSP/C amplitude on individual trials were observed, the connection was presumed to be chemical as well. The most reliable method to distinguish the electrical and chemical connections is the application of a blocker of GABAergic transmission (10 μM SR 95531). A total of 57 pair connections were recorded in the presence of gabazine, with 7 dual connections confirmed by blocking the GABAergic component of the postsynaptic response and recording the remaining electrical component (Figure 1C). Most connections were tested using standard internal solution and without synaptic blockers, which may underestimate the prevalence of very weak inhibitory synapses (IPSC amplitude < 4 pA) in the presence of strong electrical coupling (CC > 20%).

IPSC synchrony between pairs was defined as the peak of the normalized cross-correlograms (IPSC peak times, bin size = 1 ms). Spontaneous activity was measured in pairs in VC (holding potential -50 mV) for 0.5 to 2 min. Outward currents (IPSCs) were detected with a simple threshold detection algorithm. Background level was determined by shuffling the inter-event intervals and used to normalize the cross-correlograms. Traces with very low IPSC frequency were disregarded, as the baseline of the cross-correlogram was insufficient.

*Cell labeling and reconstruction*

Individual MLIs were filled with biocytin (0.5%) via the patch pipette. The whole-cell configuration was maintained for at least 15 min before pulling an outside-out patch to seal off the membrane. Slices were fixed by immersion in cold 4% paraformaldehyde solution, and kept for at least 24h. After fixation, the slices containing biocytin-filled cells were rinsed in PBS, and 0.1% Triton-X100 was used to permeabilize cell membranes. The slices were then incubated in streptavidin conjugated with Alexa 488 (Invitrogen) for 2h. Following incubation, sections were mounted between glass and coverslip in Vectashield (hard set) containing DAPI stain to visualize cell nuclei throughout the slice. Slices were then visualized using confocal microscopy. A spinning

disk confocal microscope (PerkinElmer) was used to acquire a high resolution image stack of the cell, with a lateral pixel size of 123 nm and a depth resolution of 0.3 μm.

Interneuron morphologies were manually reconstructed using the TREES toolbox in Matlab (Cuntz et al., 2011; http://www.treestoolbox.org). The reconstructed cells (n = 12) were scaled, re-aligned relative to the sagittal plane, centered with their soma at the origin, and rotated such that the y axis corresponds to the vertical axis of the molecular layer and the x axis is oriented along the Purkinje cell layer. By convention, the longer axon was directed to the left side. Shrinkage caused by fixation and slice mounting was estimated by comparing the morphology of the same cell imaged with two-photon microscopy in the live slice before fixation and with confocal microscopy after immunohistochemistry and mounted between slide and coverslip. The xy shrinkage was small; confocal/2P = 0.914 ± 0.055 (n = 45 segments). However, shrinkage in z was more substantial (confocal/2P = 0.291 ± 0.334; n = 45 segments), as expected, and therefore difficult to estimate accurately. We used the factors xy = 1.1 and z = 3.4 to correct the reconstructed confocal morphologies. The dendritic and axonal density maps were estimated as described previously (Lübke et al., 2003) for the xy plane and the yz plane. Along the transverse axis (z axis), the standard deviation of the density was $2\sigma$ = 24.1 μm for dendrites and $2\sigma$ = 41.3 μm for axons.

*Analysis and statistics*

The probabilities of electrical ($p_E$) and chemical ($p_C$) connections were determined experimentally. The probability of a connection is defined as the ratio between the total number of observed connections and the total number of possible connections. For each experimentally measured pair, there is one possible electrical connection and two possible chemical connections, therefore:

$$p_E = n_E / n_{pairs}$$
$$p_C = n_C / (2*n_{pairs})$$

where $n_E$ is the total number of electrical connections, $n_C$ is the total number of chemical connections, and $n_{pairs}$ is the total number of pairs tested.

The connectivity configuration of each recorded group (pair, triplet or quadruplet) was transformed into two graphs in the NetworkX package for Python: the electrical connectivity into an undirected graph and the chemical connectivity into a digraph with directed chemical edges.

To count the occurrence of triplet patterns, all quadruplets were divided into 4 triplets. All triplet graphs were tested for isomorphisms for each connection type individually. There are 16 different chemical triplet patterns (including bidirectional connections) and 4 different electrical triplet patterns. Error bars correspond to S.D. of a bootstrap analysis of the experimental data set (repeat = 100).

The clustering coefficient of a node i ($C_i$) is calculated as the ratio between the number of actual connections between the neighbors of node i ($n_i$) and the number of potential connections between the neighbors of node i ($p_i$) according to

$$C_i = n_i / p_i$$

The standard clustering coefficient, usually applied to entire networks (Watts and Strogatz, 1998), has been adapted here for sub-networks of triplets and quadruplets. Since the clustering coefficient of a node in a graph can only be calculated if the node receives at least 2 connections, the clustering coefficient was averaged over the configurations where *C* could be measured. For instance, for a recording of three neurons (triplet), *C* can take 3 values: 1 if it is fully connected (pattern #4, Figure 4A), 0 if it has 2 connections (pattern #3, Figure 4A), and not a number it is not measurable, i.e. if it has less than 2 connections (patterns #1 and #2, Figure 4A). As patterns with no connections do not contribute to the measure of *C*, we introduce the anti-clustering coefficient *AC*, which is calculated in the same way as the clustering

coefficient but using the complement graph. It measures how clustered the absences of connections are. For a triplet, *AC* can take 3 values: 1 if there is no connection (= 3 times "absence of connection"; pattern #1, Figure 4A), 0 if it has 1 connection (= 2 times "absence of connection"; pattern #3, Figure 4A), and not a number if it is not measurable, i.e. if it has more than 2 connections (patterns #1 and #2, Figure 4A). *C* and *AC* were evaluated separately for triplets and quadruplets and then averaged.

Chemical triplet patterns were grouped into transitive and intransitive patterns. Transitivity is a property of a directed graph G. A transitive graph G fulfills the condition that if uv and vw are directed edges in G and u ≠ w, then uw is also a directed edge in G (Bang-Jensen and Gutin, 2008). Intransitive graphs do not fulfill this condition. Graphs that do not satisfy the first part of the condition were excluded from the grouping.

The common neighbor analysis was performed in a sequential way. Estimation of significance for the comparison of probabilities between pairs with a common neighbor and other pairs was done with a $\chi^2$ test (or a Fisher's exact test for cases in which the number of occurrences was below 5), and the comparison with the non-uniform random predictions was done with Monte Carlo methods (repeat = 10,000; see below). Data are given as mean ± S.D.

*Random models of connectivity*

Specific connectivity is generally defined by comparison to equivalent random predictions. Identifying such differences can be used to rule out simple random connectivity models, but can also improve our understanding of what the actual connectivity rules are. The dependence of connection probability on intersomatic distance (Stepanyants and Chklovskii, 2005) is expected to influence the patterns of connections, in particular for higher order connectivity (Artzy-Randrup et al., 2004). We have therefore included specific parameters, such as intersomatic distance and the vertical position in the ML, and generated four models of random connectivity to compare with the

experimentally observed connectivity (Figure S3A).

The first model represents the simplest case: connections between neurons are formed independently of each other based on the connection probabilities $p_E$ and $p_C$, and independent of other parameters. This model is called the “uniform random” model, as the probabilities $p_E$ and $p_C$ are uniform with respect to distance.

The second model is called the “non-uniform random” model, as the probabilities of electrical and chemical connections are distance dependent and determined by the experimentally measured distribution of $p_E$ and $p_C$ vs. the intersomatic distance between recorded cells (Figure 2A,B). To combine the interpolation of these functions for xy distance and z distance, we perform the following approximation:

If $\Delta z \geq 30$ μm : $p_E = 0$

If $\Delta z < 30$ μm : $p_E = f_i$ (xy), interpolations (i = 1,2,3; Figure S2D)

If $\Delta z \geq 40$ μm : $p_C = 0$

If $\Delta z < 40$ μm : $p_C = f_i$ (xy), interpolations (i = 1,2; Figure S2E)

The third model is called “uniform random with ML position” model. The elements were individually determined based on the pairwise respective ML positions and using the interpolated 2-D functions $f_E$ ($ML_1$, $ML_2$) and $f_C$ ($ML_1$, $ML_2$) (Figure S3C).

The fourth model is called “non-uniform random with ML position” model. The elements were individually determined based on the pairwise difference in ML positions $ML_2$-$ML_1$ and the pairwise intersomatic distances (xy), using the interpolated functions $f_E$ (xy) and $f_C$ (xy) (Figure S3E).

For the pairwise connectivity, probabilities of observing each individual type of connection between MLI pairs were extracted from the data and compared to the predictions of the models of random connectivity. We generated 100 artificial instances of the 420 experimentally recorded pairs, for each of the

four predictions (Monte Carlo method, see below). All random connectivity models assume that connections are made independently of each other, implying:

| | |
|---|---|
| p(no connection) | $= (1-p_E) * (1-p_C)^2$ |
| p(electrical only) | $= p_E * (1-p_C)^2$ |
| p(chemical only) | $= 2 * p_C * (1-p_E) * (1-p_C)$ |
| p(chemical and electrical) | $= 2 * p_C * p_E * (1-p_C)$ |
| p(bidirectional chemical) | $= p_C^2 * (1-p_E)$ |
| p(bidirectional and electrical) | $= p_C^2 * p_E$ |

For the non-uniform random models, the intersomatic distance between each pair of neurons was used to determine and $p_E$ and $p_C$, as described above.

For the analysis of higher order connectivity, we generated 1000 artificial instances of the 65 triplets and 27 quadruplets which were experimentally recorded (Monte Carlo methods, see below). Occurrences of patterns in the data were compared to the occurrences of patterns predicted by the four random connectivity models, and measured in the same way. The non-independence of the 4 triplets extracted from a quadruplet is therefore also included in the model predictions.

The values of average clustering coefficients for the experimental data were compared to the average clustering coefficients, calculated in the same way, for the triplets and quadruplets connected according to the random connectivity models. For the average *C* and *AC* vs. z dispersion, the quadruplets were also divided into triplets. The depth differences for each element of the triplet were averaged (mean z dispersion), and triplets were grouped into bins according to their mean z dispersion. *C* and *AC* were calculated in the same way as described above, for each bin. For the two random connectivity predictions, 1000 repetitions of the experimental configuration were used and calculated in the same way as for the data.

*Monte Carlo method*

Here we describe our Monte Carlo procedure for calculating p values for the higher-order connectivity. The analysis of the pair connectivity was done analogously. The experimental dataset corresponds to a set S containing 27 quadruplets and 65 triplets. For each of the models tested (uniform, non-uniform, uniform with ML position, non-uniform with ML position), we first generated matrices $M_E$ and $M_C$ containing the probabilities of electrical and chemical connections, respectively, between all cells recorded in each configuration in S (each of the 65 triplets and each of the 27 quadruplets).

Next, we generated B random instances (B = 10 000) of the full set S. For each random instance $b \in \{1, \cdots, B\}$, we transformed the connection probability matrices $M_E$ and $M_C$ into connectivity matrices and undirected graphs $G_E$ for the electrical connectivity and directed graphs $G_C$ for the chemical connectivity. The quadruplet graphs were separated into 4 triplet sub-graphs. For each non-isomorphic pattern (k = 4 possible electrical triplet patterns and k = 16 possible chemical triplet patterns), we counted the number of occurrences $c_{i,b}$ , $i \in \{1, \cdots, k\}$. Across the B random instances of the set, we counted how many times ($p_{sup}$) the number of occurrences predicted by the model $c_{i,b}$ are superior or equal to the observed number of occurrences *c*, and how many times they are inferior or equal ($p_{inf}$):

$$p_{sup} = \{\#b, c_{i,b} \geq c\} \quad and \quad p_{inf} = \{\#b, c_{i,b} \leq c\} \qquad b \in \{1, \cdots, B\}$$

The p values for each pattern i, which determines the probability that the null hypothesis is true ($H_0$ : “There is no difference between the observed and the predicted occurrence of the pattern i”) are then given by :

$$p_i = max\left(\frac{\min(p_{inf}, p_{sup})}{B}, \frac{1}{B}\right) \; i \in \{1, \cdots, k\}$$

The formula prevents the p value from being equal to zero when $p_{sup}$ or $p_{inf}$ are equal to zero.

*Multiple hypothesis corrections*

It was necessary to correct the raw p values for multiple hypothesis testing in the case of the 6 possible connections between pairs (none, electrical only, chemical only, dual, bidirectional, full), the 4 possible electrical triplet patterns, the 16 possible chemical triplet patterns and the 3 possible connections between pairs in the common neighbor analysis. We applied the Bonferroni correction for k hypotheses, and report the corrected p values given by:

$$p_i{}^* = \min(k\, p_i, 1) \quad i \in \{1, \cdots, k\}$$

*Transitive / intransitive grouping*

We tested for the occurrence of transitive and intransitive triplet patterns, based on the signature triplet representation in other networks (Milo et al., 2004) and previous reports of the transitive property in social networks (Holland and Leinhardt, 1970) and neural networks (Nikolić, 2007). For this we grouped the 16 possible chemical triplet patterns into three groups: the ones satisfying the transitive relation; the transitive patterns (n = 4; patterns #10, #12, #14, #16), the ones not satisfying the transitive relation; the intransitive patterns (n = 7; patterns #6, #7, #8, #9, #11, #13, #15) and the ones where the first part of the transitive relation could not be applied (n = 5; patterns #1, #2, #3, #4, #5).

The grouping into transitive and intransitive patterns was originally tested with a subset of the data, after obtaining n = 8 quadruplets and n = 17 triplets. The overrepresentation of transitive triplets was significant when compared to the predictions of both the uniform and non-uniform random models. Only after this hypothesis was formulated, the second subset of the data was collected to test this hypothesis only. The n = 19 quadruplets and n = 48 triplets were used to compare with the predictions of the random models and significant differences were found when comparing with the uniform and non-uniform random models (p = 0.006 and p = 0.003 respectively).

An alternative way to assess the transitivity of complex networks is to

compute their transitivity score (Holland and Leinhardt, 1970; Milo et al., 2004). For each of the 16 triplets, the transitivity score (TS) counts the number of successful transitive relations, and the intransitivity score (IS) counts the number of unsuccessful transitive relations (Figure S5A). For instance, the pattern #16 containing the full connectivity, also called “clique”, involves 6 successful transitive relations and therefore obtains a TS of 6 and an IS of 0. The patterns for which the first part of the transitive relation could not be satisfied obtained both TS and IS of 0. Note that the patterns with an IS different from 0 are the intransitive patterns as described earlier. For the data, we calculated a total TS = 20 and IS = 15. We compared these values to the scores predicted by random connectivity models and obtained significant differences (Figure S5E). The TS is significantly higher than predicted by the random models ($p = 0.0038$ and $p = 0.0022$ for uniform and non-uniform random models, respectively) and the IS is significantly lower than predicted by the random models without ML position ($p = 0.0013$ and $p = 0.0029$ for uniform and non-uniform random models, respectively). This latter result confirms that the chemical network of MLIs has a preference for locally transitive motifs over locally intransitive motifs.

*Position of the interneurons in the molecular layer*

The positions of the recorded cells taken from the DIC images were collected and normalized according to the positions of the Purkinje cell layer (position = 0) and the pial surface (position = 1; Figure S7A). To establish the top-to-bottom orientation in the molecular layer (ML) of the transitive patterns, the ML positions of the three neurons were arranged according to their connectivity. For the feed-forward pattern (#10) the origin neuron (1) sends two diverging connections, the intermediate neuron (2) sends one connection and receives one, and the target neuron (3) receives two converging connections (Figure 8B, n = 11). For the regulating mutual pattern (#14, n = 3), the target neuron (3) receives two converging connections, and the two other neurons are reciprocally connected, which means that there is not an origin neuron defined by connectivity. The neuron highest in the ML from the two reciprocally connected ones was chosen as neuron (1). The means of the

three ML positions of all transitive patterns were significantly different (one-way ANOVA, $p = 0.0002$, $n = 14$) as well as significantly different pairwise (paired *t*-test; neuron (1) vs. (2), $p = 0.013$; neuron (2) vs. (3), $p = 0.014$; neuron (1) vs. (3) $p = 0.0001$). The average ML position of neuron (1) and (2) was also significantly different from the position of neuron (3) (paired *t*-test, $p = 0.0004$), accounting for the bias introduced by selecting neuron (1) in the regulating mutual pattern.

This result suggests an influence of the ML position on morphology and connectivity of MLIs. From the morphological point of view, it appears that stellate and basket cells are not two distinct populations but rather form a continuum, with the main parameter being the vertical position in the molecular layer (Sultan and Bower, 1998). For this reason, the connectivity of MLIs is likely to depend on the ML position. In the following analysis, we divided the vertical extent of the ML into 3 parts and grouped MLIs in their respective “third” of the ML. The probability of forming an electrical connection appeared to be related to the position of the cell in the ML (Figure S7B): $p_E = 0.46$ for MLIs in the lower third, $p_E = 0.45$ in the middle third and $p_E = 0.39$ in the upper third of the ML. For the chemical connectivity, we separated the probability of receiving a connection $p_{C\_IN}$ and the probability of sending a connection $p_{C\_OUT}$. MLIs in the upper third of the ML have a higher probability of sending $p_{C\_OUT} = 0.23$ than receiving a connection $p_{C\_IN} = 0.09$ ($\chi^2$-test, $p = 0.0003$; Figure S7B). Moreover, the probability of receiving a connection is lower in the upper third $p_{C\_IN} = 0.09$ than in the middle third $p_{C\_IN} = 0.21$ ($\chi^2$-test ; $p = 0.0004$) and than in the lower third $p_{C\_IN} = 0.25$ ($\chi^2$-test ; $p = 0.0004$). These results are in accordance with the morphologies of MLIs (Figure S7C). The main axons of MLIs tend to stay at the same level in the ML as the soma, which means that axons can predominantly target cells whose dendrites are at the same ML level. Cells in the lower third have large dendrites, some extending in the whole ML and can therefore receive connections from all other MLIs. On the other hand, cells in the upper third have short dendrites and can receive connections predominantly from other upper third cells.

Despite significant differences in connectivity when examining individual "thirds" of the ML, we conclude that the MLIs form a continuous population with gradually changing properties across the ML, as previously suggested (Rakic, 1972; Sultan and Bower, 1998; Trigo et al., 2010). We show here evidence from morphology, physiology and connectivity. First, we observed a gradual transition of morphologies from top to bottom of the ML (Figure S7C). We applied a Sholl analysis to the dendritic tree of 12 reconstructed cells and found that the length of maximal intersection is negatively correlated with the ML position (correlation coefficient r = -0.85; Figure S8A,B). Next, we observed that MLI input resistance $R_{input}$ increased with position in the ML (correlation coefficient r = 0.65; Figure S8C). This correlation can be explained in part by the decrease of the dendritic length with the ML position (Figure S8A,B). We observe that the spontaneous firing rate does not show a discontinuous change between top and bottom of the ML. Next, we find that the probability of electrical connection and the probability of receiving a chemical connection continuously decrease with ML position (linear fit, p = 0.023 and 0.007, respectively), consistent with the change in morphology. Moreover, the synaptic strength (coupling coefficient and IPSC) shows no segregation across the ML (correlation coefficients r = -0.28, r = -0.43 and r = -0.53 respectively; Figure S8F-H).

*Construction of random connectivity models using molecular layer position*

As the connectivity is evidently influenced by the ML position, we therefore constructed two additional random models including the ML position as a parameter; one model with uniform random and one with non-uniform random probability of connection with respect to intersomatic distances (Figure S3A).

First, as the probabilities of receiving and sending a connection depend on the vertical position in the ML of the neuron, it was necessary to account for the position of both cells in the pair when building a pairwise connectivity model. As the simplest approximation, we divided the ML into 3 parts and established the corresponding 3 x 3 matrix of probability of connections between cells in those 3 parts, for electrical and chemical connections (Figure S3B). As the electrical connections count as bidirectional, the matrix of the probability of electrical connections is only filled on one side. We first represented the

matrices as probability of connection as a function of the ML position of the receiving MLI (Figure S3C, left). This highlights the effect of the reduced probability of receiving a connection (electrical and chemical) the higher the cell is in the ML. The matrices were then turned into 2D interpolations to construct the “uniform random with ML position” model (Figure S3C, right).

Second, we wanted to include both the ML position and the intersomatic distances. To simplify, we used the difference in ML position instead of the full 3 x 3 connection matrix. The ML position difference was calculated as the ML position of the receiving cell minus the ML position of the sending cell, such that positive ML differences represent connections going up the ML and negative ML differences represent connections going down the ML. We observed a relationship of the probability of connection (electrical and chemical) with the difference in ML positions, with a higher probability of a chemical connection when going from top to bottom of the ML (Figure S3D). We next divided the data into two parts, one exhibiting positive and the other negative ML differences, and established the relationship of their probability of connection with intersomatic distances (Figure S3E). The chemical probability of connection indeed showed markedly different distance dependence for connections going down the ML (high probability and long tail) and connections going up (low probability, short tail). We interpolated these functions and used them for the “non-uniform random with ML position” model.

We then compared the observed connectivity with the connectivity predicted by these two new random models. All four random models are presented in Figure S3-S6 in order to facilitate the comparison. First, at the pair level, we observed that both models including ML position predict a reduced occurrence of bidirectional chemical connections (Figure S4A,B). This is consistent with the preference for chemical connections going down the ML and therefore a reduced probability of finding bidirectional connections between cells at different levels in the ML. Moreover, the underrepresentation of fully connected pairs (bidirectional chemical and electrical), significant compared to the uniform and non-uniform random models, was non-

significant compared to the random models with ML position, presumably for the same reason stated above. In this respect, the random models with ML position are better at representing the data at the pair level.

At the triplet level, we first tested the electrical connectivity, and did not observe any markedly different result compared to the random models without ML position (Figure S4C,D). As expected, the ML position does not improve the prediction of the higher clustering coefficient in the data by the random models.

We next tested the chemical connectivity at the triplet level and counted the observed and predicted occurrences of all 16 non-isomorphic triplet patterns (Figure S5B). As before, the occurrence of the feed-forward pattern (#10) is significantly different from the occurrence predicted by random models with ML position (Monte Carlo method, Bonferroni corrected p values: $p = 0.0016$ and 0.042, uniform and non-uniform random with ML position, respectively). We observed that the chemical clustering coefficient in the data remained higher than the one predicted by the random models (Figure S5C). However, when grouping into transitive and intransitive triplet patterns, we observed that the overrepresentation of transitive patterns remained, while the underrepresentation of intransitive patterns was not significant anymore when comparing to predictions of the non-uniform random model with ML position (Figure S5D; $p = 0.077$). This result was confirmed using the alternative way for assessing transitivity; determining the transitivity and intransitivity scores (Figure S5A,E). It can in part be explained by the preference for chemical connections going down the ML (Figure S7A,B). Indeed, it implies that the models including ML position have a tendency to predict a lower occurrence of loop patterns than the random models not including ML position, and therefore predict a lower level of intransitive patterns. From this perspective, we can confirm that the models of random connectivity including ML position and intersomatic distances appear to provide a better description of the actual connectivity, but are still incomplete.

Finally, we repeated the common neighbor analysis for the comparison with

the non-uniform random model with ML position (Figure S6). The main results found when comparing with the non-uniform random model were confirmed, in particular the structured overlap of the electrical and chemical networks (Figure S6B).

## Supplemental References


Artzy-Randrup, Y., Fleishman, S.J., Ben-Tal, N., and Stone, L. (2004). Comment on “Network motifs: simple building blocks of complex networks” and “Superfamilies of evolved and designed networks”. Science *305*, 1107; author reply 1107.

Bang-Jensen, J., and Gutin, G. (2008). Digraphs: Theory, Algorithms and Applications (Berlin: Springer).

Clark, B.A., and Cull-Candy, S.G. (2002). Activity-dependent recruitment of extrasynaptic NMDA receptor activation at an AMPA receptor-only synapse. J. Neurosci. *22*, 4428–4436.

Cuntz, H., Forstner, F., Borst, A., and Häusser, M. (2011). The TREES toolbox--probing the basis of axonal and dendritic branching. Neuroinformatics *9*, 91–96.

Davie, J.T., Kole, M.H.P., Letzkus, J.J., Rancz, E.A., Spruston, N., Stuart, G.J., and Häusser, M. (2006). Dendritic patch-clamp recording. Nat. Protoc. *1*, 1235–1247.

Holland, P., and Leinhardt, S. (1970). A Method for Detecting Structure in Sociometric Data. Am. J. Sociol. *76*, 492–513.

Lübke, J., Roth, A., Feldmeyer, D., and Sakmann, B. (2003). Morphometric analysis of the columnar innervation domain of neurons connecting layer 4 and layer 2/3 of juvenile rat barrel cortex. Cereb. Cortex *13*, 1051–1063.

Milo, R., Itzkovitz, S., Kashtan, N., Levitt, R., Shen-Orr, S., Ayzenshtat, I., Sheffer, M., and Alon, U. (2004). Superfamilies of evolved and designed networks. Science *303*, 1538–1542.

Nikolić, D. (2007). Non-parametric detection of temporal order across pairwise measurements of time delays. J. Comput. Neurosci. *22*, 5–19.

Rakic, P. (1972). Extrinsic cytological determinants of basket and stellate cell dendritic pattern in the cerebellar molecular layer. J. Comp. Neurol. *146*, 335–354.

Stepanyants, A., and Chklovskii, D.B. (2005). Neurogeometry and potential synaptic connectivity. Trends Neurosci. *28*, 387–394.

Sultan, F., and Bower, J.M. (1998). Quantitative Golgi study of the rat cerebellar molecular layer interneurons using principal component analysis. J. Comp. Neurol. *393*, 353–373.

Trigo, F.F., Bouhours, B., Rostaing, P., Papageorgiou, G., Corrie, J.E.T., Triller, A., Ogden, D., and Marty, A. (2010). Presynaptic miniature GABAergic currents in developing interneurons. Neuron *66*, 235–247.

Watts, D.J., and Strogatz, S.H. (1998). Collective dynamics of “small-world” networks. Nature *393*, 440–442.
